# The RNA-dependent DNA methylation pathway is required to restrict *SPOROCYTELESS/NOZZLE* expression to specify a single female germ cell precursor in *Arabidopsis*

**DOI:** 10.1242/dev.194274

**Published:** 2020-12-13

**Authors:** Marta A. Mendes, Rosanna Petrella, Mara Cucinotta, Edoardo Vignati, Stefano Gatti, Sara C. Pinto, Dayton C. Bird, Veronica Gregis, Hugh Dickinson, Matthew R. Tucker, Lucia Colombo

**Affiliations:** 1 Dipartimento di Bioscienze, Università degli Studi di Milano, Via Celoria 26, 20133 Milano, Italy; 2 LAQV REQUIMTE, Departamento de Biologia, Faculdade de Ciências, Universidade do Porto, Rua do Campo Alegre s/n, 4169-007 Porto, Portugal; 3 School of Agriculture, Food, and Wine, The University of Adelaide, Waite Campus, Urrbrae, SA 5064, Australia; 4 Department of Plant Sciences, University of Oxford, South Parks Road, Oxford OX1 3RB, UK

**Keywords:** Female germline, MADS-box transcription factors, MMC, RdDM, SPOROCYTELESS, SEEDSTICK

## Abstract

In higher plants, the female germline is formed from the megaspore mother cell (MMC), a single cell in the premeiotic ovule. Previously, it was reported that mutants in the RNA-dependent DNA methylation (RdDM) pathway might be involved in restricting the female germline to a single nucellus cell. We show that the DRM methyltransferase double mutant *drm1drm2* also presents ectopic enlarged cells, consistent with supernumerary MMC-like cells. In wild-type ovules, MMC differentiation requires SPOROCYTELESS/NOZZLE (SPL/NZZ), as demonstrated by the *spl/nzz* mutant failing to develop an MMC. We address the poorly understood upstream regulation of SPL/NZZ in ovules, showing that the RdDM pathway is important to restrict SPL/NZZ expression. In *ago9*, *rdr6* and *drm1drm2* mutants, SPL/NZZ is expressed ectopically, suggesting that the multiple MMC-like cells observed might be attributable to the ectopic expression of SPL/NZZ. We show that the ovule identity gene, *SEEDSTICK*, directly regulates *AGO9* and *RDR6* expression in the ovule and therefore indirectly regulates SPL/NZZ expression. A model is presented describing the network required to restrict SPL/NZZ expression to specify a single MMC.

## Abstract (precis)

**Highlighted Article:** The control of SPL/NZZ expression by the RdDM pathway and STK is essential for the formation of a single female germline precursor in *Arabidopsis*.

## INTRODUCTION

Plants alternate a sporophytic, diploid phase with a highly reduced gametophytic, haploid phase, during which the gametes are formed. The female germline in angiosperms initiates in the first phase of ovule development through the differentiation of a distal subepidermal cell termed the megaspore mother cell (MMC), which quickly becomes morphologically distinguishable from the surrounding sporophytic cells by its size. The MMC undergoes meiosis to form four haploid megaspores that, in *Arabidopsis*, are typically arranged in a linear tetrad. Only one of the four megaspores survives to form the functional megaspore (FM), whereas the remaining three degenerate through programmed cell death. After megasporogenesis, the FM follows the *Polygonum*-type pattern of megagametogenesis involving two mitoses without cytokinesis, resulting in a four-nucleate syncytium with two nuclei at each pole, and a third mitosis, after which cell plates are formed between all eight nuclei. Before cell plate formation, one of the nuclei from each pole migrates towards the centre of the developing embryo sac and fuses with the other to form the homodiploid nucleus of the central cell. The result is a seven-celled structure consisting of three antipodal cells in the chalazal pole, one diploid central cell, one egg cell and two synergid cells in the micropylar pole. The mature embryo sac is surrounded by two sporophytic cell layers termed integuments (Christensen et al., 1997; Yadegari and Drews, 2004).

At the molecular level, the early phases of ovule development are controlled by a number of transcription factors, hormones and small RNA-related pathways (reviewed by Pinto et al., 2019). Ovule identity in *Arabidopsis* is controlled redundantly by the MADS-box transcription factors SEEDSTICK (STK) and SHATTERPROOF 1 and 2 (SHP1 and SHP2) (Favaro et al., 2003; Pinyopich et al., 2003; Brambilla et al., 2007). STK is expressed in many sporophytic cell types, including the nucellus, chalaza and integuments, and has been shown to play key roles in several stages of ovule and seed development (Matias-Hernandez et al., 2010; Mizzotti et al., 2012, 2014; Mendes et al., 2013, 2016; Balanzà et al., 2016; Ezquer et al., 2016; Cucinotta et al., 2016; Herrera-Ubaldo et al., 2019; Di Marzo et al., 2020; Petrella et al., 2020).

After ovule initiation, the SPL/NZZ transcription factor was previously described to be essential for MMC differentiation, because in the *spl/nzz* mutant the large majority of ovules (∼99%) do not form an MMC (Yang et al., 1999; Schiefthaler et al., 1999). In contrast to *SPL*/*NZZ*, genes involved in the RNA-dependent DNA methylation (RdDM) pathway appear to limit the differentiation of multiple MMC-like cells in the premeiotic ovule. In *Arabidopsis*, DNA methylation is initially catalysed by a 24-nucleotide (nt) small interfering RNA (siRNA)-dependent RdDM pathway involving ARGONAUTE (AGO) proteins and DOMAINS REARRANGED METHYLASEs (DRM1 and DRM2). CG and CHG methylation are then maintained by DNA METHYLTRANSFERASE1 (MET1) and CHROMOMETHYLASE3 (CMT3), respectively (Law and Jacobsen, 2010). CHH methylation is maintained either through the RdDM pathway (Matzke et al., 2015) or by the CMT2 DNA methyltransferase (Zemach et al., 2013; Stroud et al., 2014). Involvement of RdDM in cell patterning in the developing ovule was proposed by Olmedo-Monfil et al. (2010), who showed that, in *Arabidopsis*, *ARGONAUTE9* (*ago9-2*) and *RNA-DEPENDENT RNA POLYMERASE6* (*rdr6-11*) mutants show additional MMC-like cells in premeiotic ovules (47 and 46%, respectively, versus 8% in wild type).

In this study, we confirmed the role of methylation in preventing the formation of multiple MMC-like cells by showing that the *drm1drm2* double mutant displays a similar phenotype to that of *ago9-2* and *rdr6-11* lines. Furthermore, we explored the possible mechanism by which the RdDM pathway is linked to MMC specification. Surprisingly, investigation of MMC specification and development in the *stk* mutant background revealed 46% of ovules to contain two or more putative MMC-like cells. Quantitative PCR confirmed that expression of *RDR6* and *AGO9* was downregulated, whereas *SPL/NZZ* was upregulated in *stk* ovules. Furthermore, chromatin immunoprecipitation (ChIP) experiments using a *STK_GFP* fusion protein showed *AGO9* and *RDR6* to be direct targets of STK. Remarkably, expression of the functional *SPL_GFP* fusion protein was found to be restricted to a few cells of the L1 layer at the tip of ovule primordium in the wild-type nucellus, but was expressed ectopically in the L1 layer of mutants representing the two major effectors of RdDM, *ago9* and *drm1drm2*. Thus, *SPL/NZZ* is expressed in the L1 layer but is required for MMC differentiation in the L2 layer; therefore, the pathway controlled by SPL/NZZ acts in a non-cell-autonomous manner, and the RdDM pathway regulates its expression. A model describing the control of female germline precursor specification in the *Arabidopsis* premeiotic ovule is proposed, bringing together data both from this study and from previous investigations.

## RESULTS

### *drm1drm2* and *stk* mutants show additional MMC-like cells

**Fig. 1.**
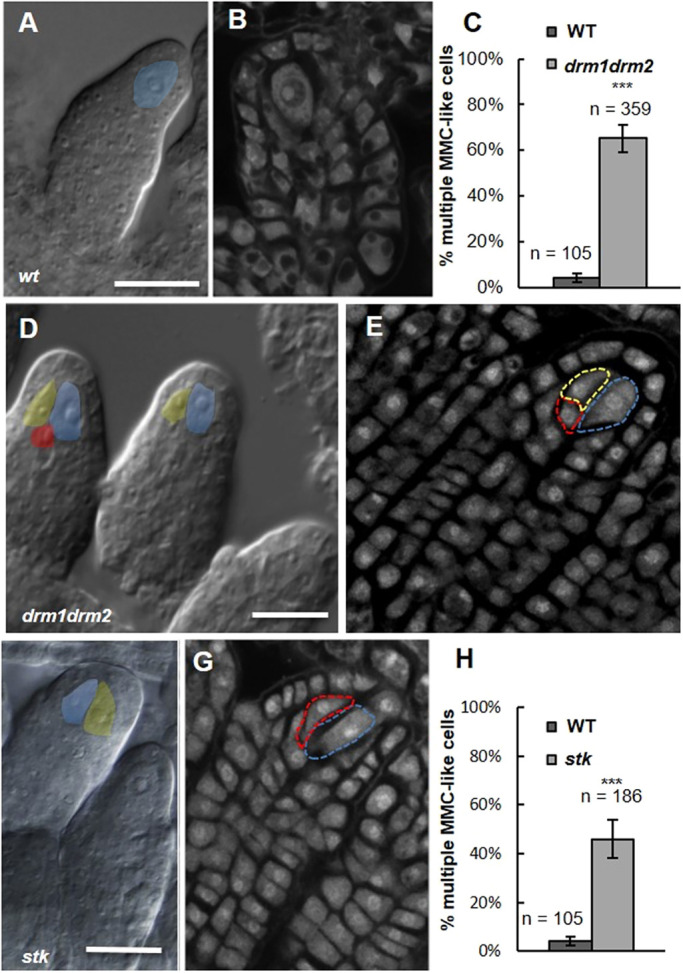
***drm1drm2* and *stk* mutants present multiple MMC-like cells in** **premeiotic** **ovules.** (A,B,D-G) DIC and confocal imaging of premeiotic ovules; in the wild type (A,B), a single enlarged cell is detected in the nucellus, corresponding to the MMC (in light blue); multiple enlarged cells were detected in the nucellus in *drm1drm2* double mutant (D,E) and in the *stk* single mutant (F,G). Scale bars: 15 µm in A,D,F. Different colours indicate the multiple enlarged cells. Confocal sections were acquired after Feulgen staining. (C,H) Graphical representation of *drm1drm2* (C) and *stk* (H) multiple nucellar enlarged cells. ***P<0.001 by Student's unpaired *t*-test; wild-type premeiotic ovules were screened as the control.

Differentiation of the MMC is of pivotal importance for the progression of female germline development, and specification of a single MMC is under strict molecular control. In the *ago9-2* mutant, it has been described that ∼47% of the ovules develop more than one MMC-like cell (Olmedo-Monfil et al., 2010). It is known that the RdDM pathway can regulate gene expression via AGO-mediated mRNA degradation or via cytosine DNA methylation of target genes by DRM1/DRM2 methylases (Matzke and Mosher, 2014). Therefore, we analysed MMC differentiation in the double *drm1drm2* mutant by differential interference contrast (DIC) microscopy clearing and Feulgen staining (confocal analysis; Braselton et al., 1996). Approximately 65% (*n*=359) of the *drm1drm2* premeiotic ovules displayed multiple MMC-like cells (Fig. 1C-E) when compared with the wild type (Fig. 1A,B), showing that the full RdDM pathway, including cytosine methylation, is pivotal for the specification of a single MMC. The formation of an MMC in the nucellus is part of the basic ovule developmental programme, which is heavily influenced by ovule identity genes, such as *STK*. Examination of premeiotic ovules in *stk* lines showed double MMC-like cells to be present in 46% of ovules (*n*=186), which was significant when compared with the wild-type situation (Fig. 1F-H). Significantly, this level of multiple MMC development is broadly similar to that reported by Olmedo-Monfil et al. (2010) for *ago9-2 *and* rdr6-11* lines and reported here for *drm1drm2* ovules. More images of the ovule primordium for each mutant line are presented in Fig. S1. We have also counted the number of fertilized and unfertilized ovules in the mature siliques of *stk* and *drm1drm2* mutants and found that wild type and *stk* produced similar ratios, whereas ∼18% of ovules were unfertilized in *drm1drm2* siliques (Fig. S2).

### RdDM gene expression is directly regulated by STK

To identify a possible connection between STK and RdDM pathway components, such as *RDR6*, *AGO9*, *DRM1* and *DRM2*, we performed real-time PCR with RNA extracted from wild-type and *stk* mutant inflorescences (Fig. S3). Quantitative real time-PCR (qRT-PCR) was used to assess the relative level of gene expression in the *stk* mutant compared with the wild type. This confirmed that *STK* was significantly downregulated in the *stk* background, consistent with previous reports (Pinyopich et al., 2003) (Fig. S3). Transcript levels of *AGO9*, *RDR6* and *DRM1* also showed significant downregulation, although *DRM2* expression was unchanged compared with the wild type (Fig. S3).

**Fig. 2.**
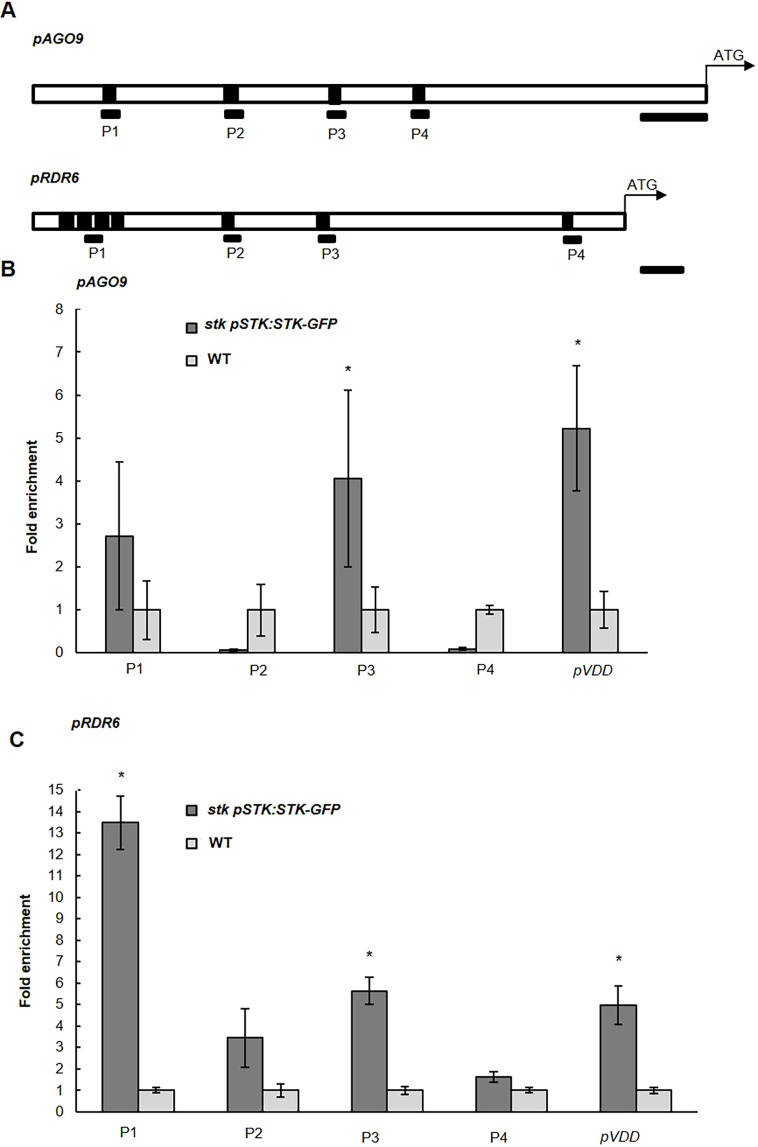
**STK directly binds the regulatory region of RDR6 and AGO9.** (A) Schematic diagram of the *AGO9* and *RDR6* putative promoter and 5′ UTRs, respectively, indicating the regions analysed by ChIP (black bars) followed by qRT-PCR; each black box represents a CArG box. Scale bars: 350 bp in top panel; 50 bp in bottom panel. (B,C) Quantitative real-time PCR analysis of ChIP assay using chromatin extracted from *stk* mutant complemented with *pSTK::STK_GFP* and wild type (as a negative control), testing the CArG box regions on *pAGO9* (B) and *pRDR6* (C); the promoter region of *VDD* was tested as the positive enrichment (Matias-Hernandez et al., 2010). For the immunoprecipitation, commercial antibodies against GFP were used. Error bars represent the propagated error value using three replicates. ChIP results of one representative experiment are shown. Positive binding site fragments were considered only if they were enriched compared with the controls in at least three independent experiments. *P<0.05 by Student's unpaired *t*-test compared with the wild type.

To assess the possibility that STK might directly regulate expression of the RdDM pathway components, we performed ChIP experiments using an *STK_GFP* line (Mizzotti et al., 2014) followed by qRT-PCR. We searched for CArG boxes (DNA binding regions recognized by the MADS-box transcription factors) in the putative regulatory regions of *RDR6*, *AGO9*, *DRM1* and *DRM2*, allowing a maximum of two mismatches in the consensus sequence (as described by Mendes et al., 2013). In the putative promoter and 5′ untranslated region (5′ UTR) of *RDR6*, seven CArG boxes were identified and subsequently divided into four regions; two of these were enriched (P1 and P3; Fig. 2A,C) in *STK_GFP* immunoprecipitated chromatin when compared with wild-type chromatin. In the *AGO9* promoter and 5′ UTR, we identified six putative CArG boxes that were divided into four regions; we could confirm an enrichment in region P3 (Fig. 2A,B). Using the same criteria, in the case of *DRM1* and *DRM2*, no CArG box-like sequences were identified in the promoter regions or in the 5′ UTR that were suitable for analysis. Taken together, these experiments suggest that STK is a direct regulator of *RDR6* transcription with two enriched regions, and an activator of *AGO9* with one region enriched.

### The identity of putative ectopic MMC-like cells in *stk* and RdDM mutants

**Fig. 3.**
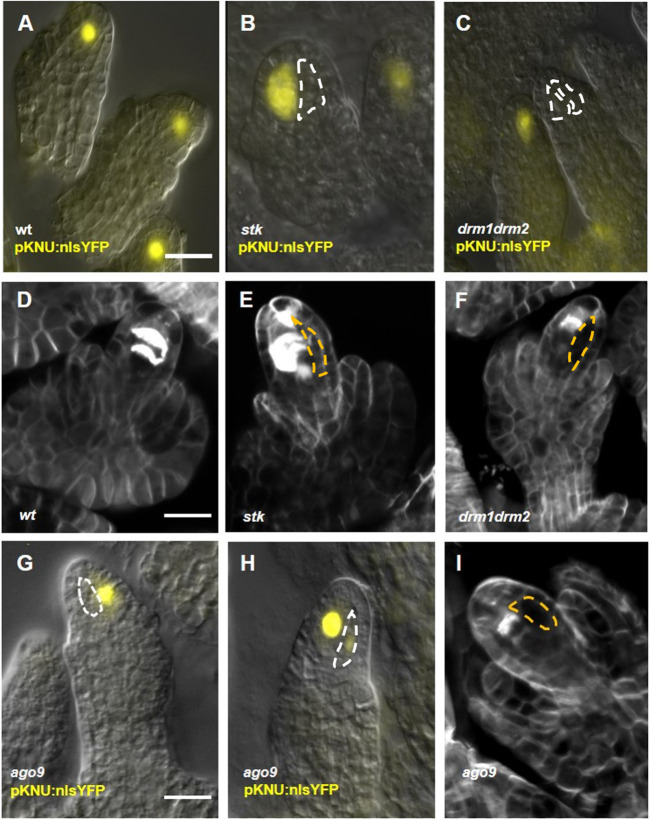
***pKNU:nlsYFP* expression analysis and meiosis staining.** (A-C,G,H) *pKNU:nlsYFP* marker in wild type (A), *stk* (B), *drm1drm2* (C) and *ago9-2* (G,H). Some examples are provided. In all the mutant backgrounds analysed, although two enlarged cells can be observed, only one was positive for YFP expression (B,G); also, several ovules did not show any YFP signal (C). (D-F,I) Callose staining in the wild type, *stk*, *drm1drm2* and *ago9* background, respectively; the analysis showed that only one cell enters meiosis. Scale bars: 15 µm in A,D,G. Dashed lines indicate the extra enlarged cells.

The *pKNU::nlsYFP* transcriptional marker specifically marks cells possessing MMC identity (Tucker et al., 2012; Fig. 3A) and was therefore used to define the identity of the supernumerary MMC-like cells in *stk* and RdDM mutants. To enable accurate comparisons, the number of ovules/cells showing yellow fluorescent protein (YFP) fluorescence in each mutant background was compared with wild-type segregants from the same cross. In *drm1drm2* and *stk* mutants, two patterns were observed in each ovule: one of the two putative MMCs expressed the YFP marker, or none of the putative MMCs expressed the marker (Fig. 3B,C). Indeed, in *stk* only 62% of the analysed ovules (*n*=468) were positive for YFP, whereas in wild-type segregants 82% of the ovules showed YFP expression (*n*=452), a statistically significant difference (*P*>0.01, Student's unpaired *t*-test; Table S1). A similar pattern was observed for *drm1drm2* double mutant ovules, whereby 61% (*n*=572) generated YFP signal compared with 94% in the wild type (*n*=152); again this difference was statistically significant (*P*>0.01, Student's unpaired *t*-test; Table S1). Interestingly, 80% (*n*=350) of *rdr6-11* mutant ovules showed YFP signal in only one of the multiple MMC-like cells, whereas the remaining ovules were negative. This indicates that in the analysed *drm1drm2*, *stk* and *rdr6* mutants, only one of the two (or more) MMC-like cells acquires MMC identity, and in some cases the wild-type MMC loses aspects of its identity (as determined by the *KNU* promoter). In *ago9-2* mutant lines, only one cell expressed YFP signal in 91% (*n*=683) of ovules, whereas in wild-type segregants 93% of the ovules (*n*=688) showed expression. Interestingly, this mutant did generate signals in more than one cell, with the second enlarged cell emitting a very faint signal in 2% of the cases; however, a similar situation was also found in the rare wild-type ovules showing multiple putative MMC-like cells, where 2% of total MMCs presented the double signal (Fig. 3G,H). The analysis of *pKNU:nlsYFP* is summarized in Table S1.

Although the extra MMC-like cells failed to activate the *KNU* promoter, we also asked whether they shared physical features with the MMC and/or whether they entered meiosis. Callose accumulates in the MMC cell wall before meiosis (Fig. S4), and during meiosis it accumulates in the walls of the four megaspores with a specific pattern. Thus, MMC expansion and progression of meiotic division was observed by assessing the pattern of callose deposition before (Fig. S4) and after meiosis (Fig. 3) in *stk*,* ago9*, *rdr6* and *drm1drm2* with respect to wild type. Aniline Blue staining highlighted remarkably similar staining patterns in the MMC wall of wild-type (Fig. S4A), *stk* (Fig. S4B), *ago9* (Fig. S4C), *rdr6* (Fig. S4D) and *drm1drm2* (Fig. S4E) premeiotic ovules (*n*>200 for each background), and in no cases was callose detected in more than one enlarged cell. During meiosis in wild-type ovules (Fig. 3D), it was possible to clearly distinguish the formation of two defined septa that are correlated with the second meiotic division (*n*=20). In *stk* and *drm1drm2*, as in *ago9-2 and rdr6-11* mutants, only one of the two enlarged cells underwent meiosis (*stk n*=15, *drm1drm2 n*=18, *ago9*-2 *n*=10 mutant and *rdr6-11 n*=10; Fig. 3E,F,I).

**Fig. 4.**
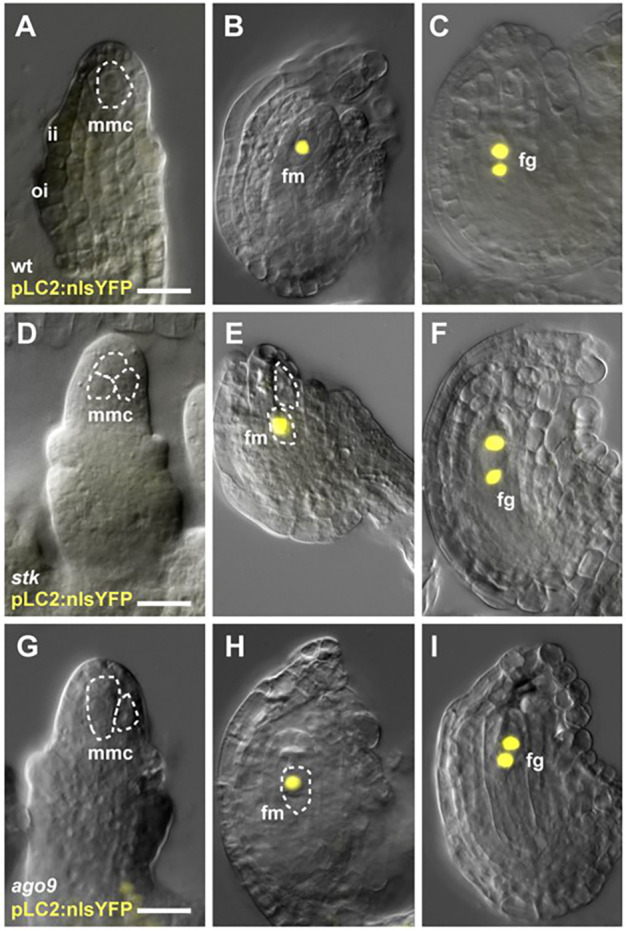
***pLC2::nlsYFP* expression analysis.** (A-C) Wild type. (D-F) *stk*. (G-I) *ago9-2*. Ovules were examined for *pLC2:nlsYFP* expression at the MMC stage (A,D,G) and after meiosis (B,C,E,F,H,I). Expression patterns were generally indistinguishable among the wild type, *ago9-2* and *stk*, despite the presence of more than one MMC-like cell in the mutant backgrounds. fg, female gametophyte; fm, functional megaspore; ii, inner integument; mmc, megaspore mother cell; oi, outer integument. Dashed lines highlight the mmc, fm or ectopic enlarged cells. Scale bars: 15 µm.

Previous examination of the RdDM pathway mutants suggested that the supernumerary MMC-like cells might represent additional functional megaspores (Olmedo-Monfil et al., 2010). In particular, it was shown that *pFM1::GUS* and *pFM2::GUS* were expressed in a single FM/female gametophyte in wild-type ovules but accumulated in multiple cells in the *ago9* mutant (Olmedo-Monfil et al., 2010). Thus, we considered the possibility that the supernumerary MMC-like cells in *stk* ovules might express a functional megaspore marker gene. To test this, we used the *pFM1::GUS* marker and found that in the majority of ovules, expression appeared to be restricted to a single FM/female gametophyte in both wild type and *stk* (Fig. S5A-E). However, diffusion of the β-glucuronidase (GUS) signal was difficult to distinguish from possible expression in extra cells. To overcome this, we used the fluorescent *pLC2:nlsYFP* marker (Tucker et al., 2012) which, in the wild type, is undetectable in the MMC but later shows expression in the functional megaspore and during the first mitotic divisions of megagametogenesis (97% positive signal, *n*=344; Fig. 4A-C). The *ago9-2* mutant, which was previously reported to show ectopic expression of the *pFM2::GUS* functional megaspore marker (Olmedo-Monfil et al., 2010), was included as a representative of the RdDM pathway. No *pLC2:nlsYFP* expression was detected in ectopic enlarged cells in *stk* or *ago9-2* mutants at the MMC stage (*n*>200; Fig. 4D,G), suggesting that these cells do not exhibit FM identity in premeiotic ovules. Surprisingly, after meiosis, expression in both mutants remained restricted to a single functional megaspore (Fig. 4E,H), and subsequently, in the two nuclei produced after the first mitotic division (Fig. 4F,I). The pattern of expression was similar to that observed in wild-type ovules and showed similar staining efficiency (*stk*, 96%, *n*=320; *ago9*-*2*, 95%, *n*=440). The only variation in pattern was detected in <2% (nine of 440) of the *ago9-2* ovules where two sources of nuclear YFP signal were detected before FM division (Fig. S6). However, it was unclear whether this signal represented *pLC2:nlsYFP* expression in one functional megaspore and one supernumerary enlarged cell or in two megaspores within the meiotic tetrad. Taken together, these data indicate that the supernumerary enlarged cells in *stk* are unlikely to possess functional megaspore or female gametophyte identity. Moreover, inactivation of the key RdDM effector, *AGO9*, leads to only subtle changes in *pLC2:nlsYFP* expression and appears insufficient to activate the functional megaspore programme fully in the extra enlarged cell.

### Ectopic expression of *SPL/NZZ* induces multiple MMC-like cells

Although the supernumerary MMC-like cells in *stk* and the RdDM mutants appear unable to adopt MMC or FM identity fully, we hypothesized that their presence in the nucellus of premeiotic mutant ovules might be attributable to ectopic expression of *SPL/NZZ*, which is required for MMC initiation (Yang et al., 1999; Schiefthaler et al., 1999). Therefore, we analysed *SPL/NZZ* expression by qRT-PCR in the *stk* mutant and in the RdDM mutants *ago9*, *drm1drm2* and *rdr6* (Fig. S7A). We showed a consistent upregulation of *SPL/NZZ* transcripts in closed flowers during early stages of development in *rdr6*, *drm1drm2* and *ago9* mutants. Furthermore, when *SPL/NZZ* expression was analysed in individual flowers, enriching the first phases of megasporogenesis, we could detect a significant increase in *SPL/NZZ* expression in the *stk* mutant when compared with the wild type. Taken together, these results provided further support for the hypothesis that the presence of extranumerary MMC-like cells in the analysed mutants might be caused by an upregulation of *SPL/NZZ* transcript (Fig. S7B).

To study the spatial expression pattern of SPL/NZZ in the *stk* and RdDM mutant ovules, the activity of the *SPL/NZZ* promoter was investigated (Fig. S8) using a *pSPL5′::GUS:3′* construct. When wild-type lines were analysed, the promoter was seen to be active in the L1 cell layer of the extreme nucellar tip (white asterisks in Fig. S8) of premeiotic ovules, whereas in *stk*, *ago9-2* and *drm1drm2* lines, the construct was expressed ectopically throughout the distal nucellar primordium (Fig. S8). In order to investigate whether the upregulation of *SPL/NZZ* in the *stk* mutant was attributable to a direct regulation by STK, we analysed the regulatory region of *SPL/NZZ*, searching for CArG boxes. Two CArG boxes were detected, with a maximum of two mismatches in the consensus sequence, and grouped in two regions: P1, upstream from the coding sequence of *SPL/NZZ*; and P2 in the 3′ untranslated region (3′ UTR), as already reported by Ito et al. (2004) (Fig. S9A). When binding of STK was tested by ChIP, neither of the two regions showed enrichment in STK_GFP immunoprecipitated chromatin when compared with the wild type (Fig. S9B).

**Fig. 5.**
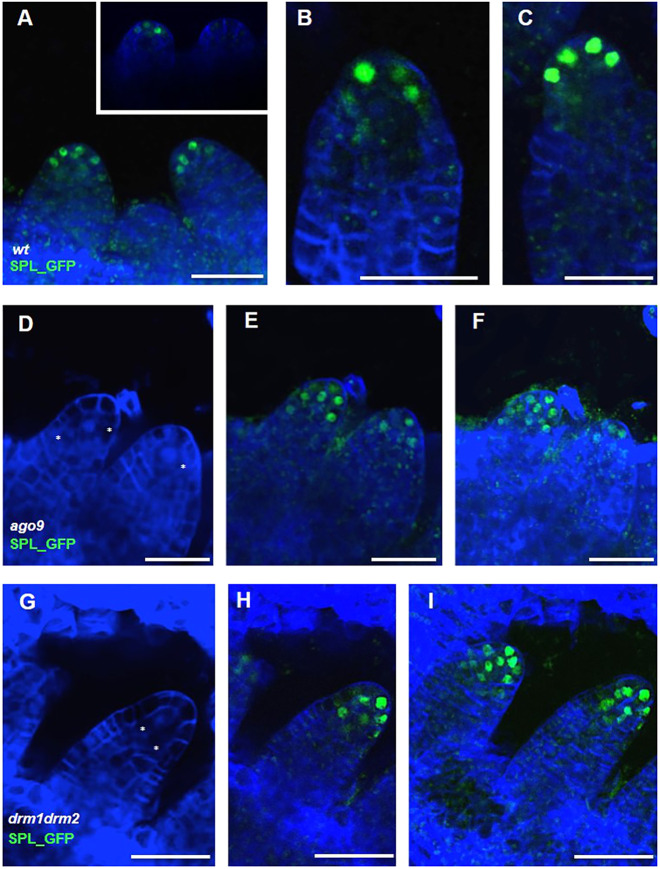
***SPL_GFP* expression analysis.** Analysis of SPL/NZZ protein localization using *pSPL_5′UTR::SPL-GFP_3′UTR*. (A-C) In the wild type, SPL expression is confined to the tip of the ovule primordium/L1 layer and premeiotic ovules. (A) Detail of ovule primordium emergence. (D) Renaissance staining of *ago9-2* ovule primordium. (E) Single stack of *SPL_GFP* in *ago9-2*. (F) *Z*-stack projection of E. (G) Renaissance staining of *drm1drm2* ovule primordia. (H) Single stack of *SPL_GFP* in *drm1drm2*. (I) *Z*-stack projection of H. The SPL expression domain was expanded to the lower layers of the nucellus in the analysed mutants. Scale bars: 15 µm in A-I. Renaissance staining was used to mark the cell walls and the nuclei (white asterisks) blue in the pictures.

To determine the location of the SPL/NZZ protein in wild-type and mutant contexts, we cloned the genomic region of *SPL* fused with GFP plus the regulatory region described above, creating *pSPL5′::SPL_GFP:3′* (*SPL_GFP)*. To verify the function of the recombinant protein, we transformed *SPL_GFP* into the *spl/nzz* mutant and confirmed that it complemented the fertility defects fully (Fig. S10). Subsequently, we analysed the expression of *SPL_GFP* in three independent lines and found that in premeiotic ovules, GFP was confined to nuclei of the L1 layer in the tip of the nucellus, similar to what was detected with the *pSPL5′::GUS:3′* transcriptional reporter. *SPL_GFP* signal was not detected in the MMC, its products or its precursors, providing compelling evidence that although SPL/NZZ is expressed in L1 cells, it is required for MMC differentiation in the L2 layer (Fig. 5A,B). *SPL_GFP* was then introgressed into mutants of the two major RdDM pathway components (*ago9-2* and *drm1drm2*) to assess any changes in expression. Analysis of ovules confirmed that the recombinant protein was localized in nuclei and exhibited the same expanded pattern shown by GUS driven by the *SPL* promoter (*pSPL5′::GUS:3′*). However, subtle differences were observed in SPL/NZZ expression between the two mutant backgrounds. In an *ago9-2* ovule that presented with an extra MMC-like cell in the nucellus of the ovule primordia, the *SPL_GFP* fusion protein was detected in more L1 layer cells when compared with the wild type (Fig. 5E). Importantly, these differences were not observed in *ago9-2* ovules, in which only one cell was enlarged (i.e. the MMC, Fig. 5D-F), as seen by comparing the number of GFP-positive nuclei in the confocal *z*-projection. In the *drm1drm2* mutant (Fig. 5G-I), SPL/NZZ protein was again detected in more L1 layer cells when compared with the wild-type situation, and the two ovules in the *z*-projection presented a clear difference in the number of nuclei containing GFP (Fig. 5I). We can conclude that the RdDM and DNA methylation pathways play an important role in the precise regulation of SPL/NZZ expression and activation in the L1 nucellar layer. During meiosis, SPL/NZZ expression remains in the cell layers surrounding the MMC (Fig. S11).

**Fig. 6.**
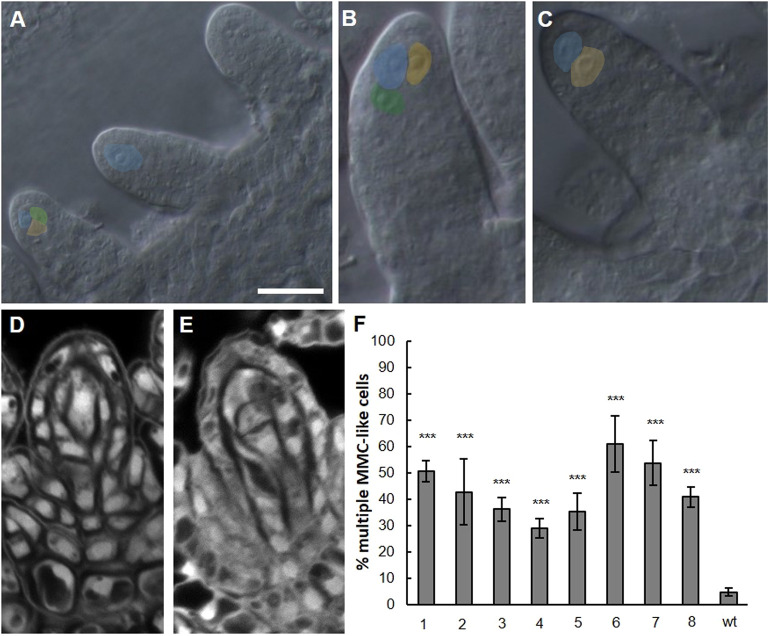
**Constitutive expression of *SPL/NZZ* induced the formation of multiple MMC-like cells.** (A-E) DIC microscopy (A-C) and confocal microscopy (D,E) of *35S::SPL/NZZ* lines. Multiple enlarged cells can be observed in the premeiotic ovule nucellus, highlighted with different colours. Confocal sections were acquired after Feulgen staining. Scale bar: 15 µm in A (for A-C). (F) Graphical representation of the percentage of multiple enlarged cells in eight *35S::SPL/NZZ* independent lines. ***P<0.001 by Student's unpaired *t*-test. Wild-type premeiotic ovules were screened as the control.

The ectopic expression of SPL/NZZ in *ago9* and *drm1drm2* mutants suggested that this might contribute to the formation of extra MMC-like cells in premeiotic ovules. To assess this hypothesis, we expressed *SPL/NZZ* under the control of the 35SCaMV promoter, which accumulates to high levels in multiple tissues, including the nucellus of premeiotic ovules. Eight independent lines were obtained in the T1 generation (Fig. 6), all of which presented multiple MMC-like cells in premeiotic ovules, as shown in Fig. 6F. The supernumerary enlarged cells in the nucellus were observed after clearing and DIC microscopy (Fig. 6A-C) and with confocal imaging after Feulgen staining (Fig. 6D,E). This observation supports the hypothesis that in *stk* and in the RdDM mutants, the formation of extra germ line precursors is caused by broader SPL/NZZ expression.

## DISCUSSION

### Absence of methylation via RdDM leads to a multiple MMC-like cell phenotype

The aim of this study was to contribute to understanding of the gene network controlling formation of a single female MMC in the young ovule of *Arabidopsis*. Olmedo-Monfil et al. (2010) reported both *ago9-2* and *rdr6-11* lines to generate additional MMC-like cells in premeiotic ovules, implicating RdDM in MMC specification. *AGO9* and *RDR6* are involved in the processing of siRNAs directing the cytosine methylation of complementary DNA (cDNA) sequences. To determine whether *AGO9-* and *RDR6*-dependent siRNAs might be involved in silencing of target sequences via DNA methylation, we investigated the role of DRM1 and DRM2, two methyltransferases that are involved solely in methylation via RdDM (Law and Jacobsen, 2010). Here, we showed that ∼65% of premeiotic ovules of *drm1drm2* lines contained multiple MMC-like cells, confirming that RdDM of target sequences is required for the specification of a single MMC in *Arabidopsis*. This finding is consistent with previous studies showing that a reduced level of methylation accompanies MMC differentiation (Ingouff et al., 2017). Similar to the MMC, a reduction in methylation has been reported during early microspore mother cell differentiation in the anther (Walker et al., 2018). DRM1 and DRM2 are also important for setting the correct methylation poise after fertilization, and DRM2 has been shown to complex with the RdDM effector AGO4 (Zhong et al., 2014) and to be expressed mainly in the developing embryo (Havecker et al., 2010), whereas both methyltransferases have been shown to be required for embryogenesis (Chow et al., 2020).

### RdDM in the ovule is regulated directly by the key ovule identity MADS-box transcription factor STK

Our data also indicate that the female developmental programme in *Arabidopsis*, including MMC specification, is coordinated by STK, a MADS-box transcription factor already implicated in a wide range of processes, including the establishment of ovule identity (Matias-Hernandez et al., 2010; Mendes et al., 2016). The formation of two or more MMC-like cells in ∼46% of the premeiotic ovules in *stk* mutants strikingly resembles the phenotype of the *ago9-2* and *rdr6-11* plants reported by Olmedo-Monfil et al. (2010) and that of our *drm1drm2* double mutants. We therefore searched for a link between STK and the RdDM pathway, finding that transcription of both *RDR6* and *AGO9* might be activated directly by STK, which we show to bind to different CArG box regions. This is consistent with the requirement of MADS-box transcription factors to bind at least to two binding sites to activate transcription (Mendes et al., 2013). Strikingly, the expression of both *AGO9* and *RDR6* is downregulated in *stk* mutant lines, confirming the functionality of these binding events.

### *SPL/NZZ* expression is restricted to a few cells in the nucellus by the STK/RdDM pathway

Previous reports revealed that 99% of premeiotic *spl/nzz* ovules fail to develop an MMC (Yang et al., 1999; Wei et al., 2015), a phenotype diametrically opposed to that of the *rdr6*, *ago9*, *drm1drm2* and *stk* plants. *SPL/NZZ* thus constitutes a strong candidate target of the STK/RdDM pathway, and the qRT-PCR data described in our paper show *SPL/NZZ* to be overexpressed in all RdDM and *stk* mutant lines. In wild-type plants, *SPL/NZZ* is expressed solely in the tip ovule primordium L1 layer cells at very early stages, as clearly shown in both *pSPL5′::GUS:3′* and *pSPL5′::SPL_GFP:3′* lines. Importantly, although both promoter expression and SPL/NZZ protein synthesis occur in these few distal cells, expression of the SPL/NZZ protein fusion was never detected in the single cell (in wild-type lines) destined to assume MMC identity, indicating that SPL/NZZ regulates MMC development in a non-cell-autonomous manner. By contrast, striking ectopic expression of *pSPL5′::GUS:3′* occurs in the L1 layer of primordial ovules of *stk*, *ago9-2* and *drm1drm2* lines; furthermore, similar ectopic expression was also detected in these cells when *ago9* and *drm1drm2* mutant plants were transformed with the *pSPL5′::SPL_GFP:3′* construct, confirming the presence of the SPL/NZZ protein*.*

Further confirmation that restriction of *SPL/NZZ* expression to only a few cells of the primordia ovule L1 layer is required for single MMC specification comes from analysis of transgenic plants expressing *SPL/NZZ* driven throughout the ovule primordium by the 35CaMV promoter. Here again, multiple MMC-like (germline precursors) cells are formed. As evidence is accumulating that the expression pattern of *SPL/NZZ* in the ovule is regulated by RdDM-mediated methylation, a sensible next step will be to determine whether the ovular SPL/NZZ is itself a target for methylation. Evidence from global methylome analysis (Zhang et al., 2006) suggests that the *SPL/NZZ* sequence is unmethylated, but it is unclear whether the sequencing system used would have detected differences in methylation at a local cellular level. If the *SPL/NZZ* genomic locus remains unmethylated in the ovule, an intermediate regulatory factor is likely to serve as a direct target of the RdDM pathway.

### *SPL/NZZ* is sufficient for germline precursor specification, but not for MMC function

The observation that extra MMC-like cells in *stk*, *ago9* and *drm1drm2* lines fail both to express an MMC identity gene and to enter meiosis indicates that although SPL/NZZ is required for initial MMC specification (i.e. germline precursor formation), it is insufficient to confer full MMC identity and function. For example, little or no expression of the MMC marker *pKNU::nlsYFP* (Tucker et al., 2012) was detectable in in the supernumerary enlarged cells of the *stk*, *ago9* and *drm1drm2* ovule primordia. Also, the functional megaspore marker *pLC2:nlsYFP*, which is expressed in the postmeiotic megaspore of wild-type plants, labels only a single cell in *stk* and *ago9* ovules, indicating that the supernumerary cells formed in these lines are unlikely to be functional megaspores. Furthermore, only one of the cells in *stk* and *drm1drm2* ovules enters meiosis, as has already been shown for *ago9* and *rdr6* mutants (Olmedo-Monfil et al., 2010). In view of the disruption of early ovule development, the observation that fertility is unaffected in *stk* mutants is surprising, and fertility was only slightly reduced (by 18%; *P*<0.01) in *drm1drm2* lines. The observation that the *KNU* promoter is inactive in 40% of the ovules of fertile *stk* and *drm1drm2* mutants (data in Table S1) is perplexing and might indicate that that *KNU* expression, despite being a feature of wild-type development, is not essential for entry into meiosis. Taken together, our data confirm that although the STK/RdDM/SPL pathway specifies the formation of a single female germline precursor, further factors are required for complete MMC function.

### STK-mediated RdDM restriction of *SPL/NZZ* expression, and a new model for female germline precursor formation

SPL/NZZ acts as an adaptor-like transcriptional repressor in *Arabidopsis*, potentially forming a bridge between TOPLESS (TPL)/related (TPR) proteins and CIN-like TCP transcription factors (Wei et al., 2015). When bound to SPL in this way, TPL proteins are proposed to repress TCP activity to promote MMC development (Wei et al., 2015) and, in the absence of SPL/NZZ, overexpression of TCP genes is proposed to result in failure of megasporogenesis and abnormal ovule development. However, constitutive repression of TCP sequences alone is not sufficient for FM development, pointing to the involvement of additional factors (Wei et al., 2015). Thus, in the ovule primordia of RdDM mutant lines, it is presumably the expansion of ectopic SPL/NZZ expression into an extended field of L1 cells that results in development of subtending supernumerary MMC-like cells (female germline percursors). Why the ectopic SPL/NZZ expression fails to develop additional fully functional MMCs is unclear. Our observation of multiple MMC-like cells being formed when SPL is expressed throughout the ovule confirms that SPL expression itself does not inhibit MMC-like cell formation, as occurs in some lateral control systems. However, the L1 is a highly specialized cell layer with a transcriptome very different from the L2, and it might simply be that additional factors necessary for MMC formation are absent from these cells. Previously, it was suggested that SPL is involved in auxin homeostasis, because in the *spl/nzz* mutant, PIN1 (auxin transporter) expression was compromised, suggesting that SPL is important for PIN1 expression (Bencivenga et al., 2012). Auxin accumulates at the tip of the nucellus, similar to the expression pattern of SPL/NZZ protein, and this interaction could be linked to MMC specification (Ceccato et al., 2013). SPL/NZZ has also been proposed to act in concert with the homeodomain transcription factor WUSCHEL (WUS), to promote MMC differentiation. Indeed, the *wus* mutant lacks a primary female germline cell in ∼12% of ovules. However, it is still unknown how the two transcription factors interact (Groß-Hardt et al., 2002). *WUS* and/or hormone-related pathways might be related to the full MMC specification.

**Fig. 7.**
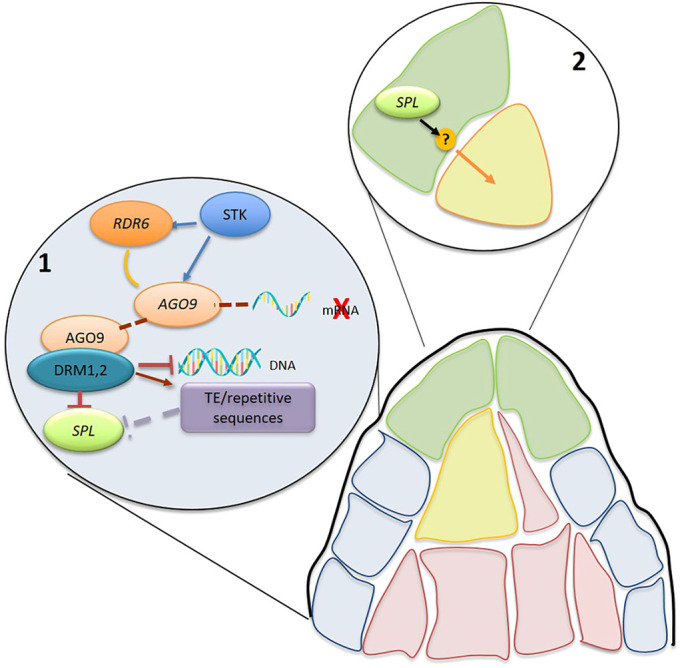
**Proposed mode of action.** In the lower part of the L1 layer, STK binds directly to *AGO9* and *RDR6* promoter regions, prompting the RdDM pathway, which finishes on silencing transposable elements or repetitive sequences that, on their way, silence *SPL/NZZ*. The RdDM pathway can also silence *SPL/NZZ* directly, by degrading its mRNA through *AGO9* and/or by methylation of the *SPL/NZZ* genomic region through DRM1 and DRM2 methyltransferases, which will result in repression. Altogether, this leads to a direct/indirect repression of *SPL* transcription in the nucellar cells, except in the apical nucellar cells (L1). In the apical nucellar cells, SPL/NZZ can promote cell expansion/elongation by activation/repression of a factor secreted from the upper cells that generates a signal for cell expansion in the cell below.

In an attempt to integrate our data with the findings of Olmedo-Monfil et al. (2010), we have developed a new model (Fig. 7) for the genetic and epigenetic control for specification of the single MMC in *Arabidopsis*. The model proposes that STK activates RdDM gene expression in the lower L1 layer cells, and siRNAs present in those cells act via either mRNA cleavage/repression or methylation to suppress SPL/NZZ expression and synthesis. Nonetheless, SPL/NZZ is synthesized and expressed only at the tip of the ovule primordium/L1 layer. The specification of a female germline precursor can be achieved via a number of possible mechanisms, presumably based on the movement of an effector molecule, possibly through the plasmodesmata, to the subtending L2 cell to initiate MMC expansion and eventual MMC specification. The nature of this effector, its mode of operation and its target(s) in the L2 cell remain to be determined. Interestingly, it has been reported that mutations in homologues of DRMs and AGO genes in rice and maize influenced MMC differentiation and/or the determination of the female germline precursor (Singh et al., 2011). Overall, this evidence suggests that our model might also be extended to crops. Greater comprehension of the mechanisms determining MMC specification could have an important role in future noteworthy crop improvement.

## MATERIALS AND METHODS

### Plant material and growth conditions

All plants of the *Arabidopsis thaliana* ecotype Columbia-0 (wild type), mutants and marker lines (Table S2) were grown in soil obtained from a ratio of 5:1:1 potting soil:vermiculite:perlite, respectively. Plants were grown first at 21°C/18°C in short-day conditions (SD; 8 h light/16 h dark) until the bolting of the rosette leaves and then in long-day conditions (LD; 8 h dark/16 h light) until flowering, with 70% humidity in both periods.

### Generation of marker lines in different mutant backgrounds

The *pKNU:nlsYFP* marker is expressed in the MMC in the premeiotic ovules. The characterized *pKNU:nlsYFP* Col wild type was crossed with *stk*, *ago9*, *rdr6* and *drm1drm2* mutants, and at least three homozygous F3 plants were analysed for expression in each combination. The *pLC2:nlsYFP* marker (pAt5g40730:nls-vYFP) is expressed in the functional megaspore and during subsequent stages of megagametogenesis in the Columbia background (Tucker et al., 2012). The characterized *pLC2:nlsYFP* Col wild-type line was crossed with *ago9* and *stk* mutants, and three homozygous F3 plants were analysed for expression in each combination.

### RNA extraction

Gene expression was evaluated in the closed flower up to stage 15 (according to Smyth et al., 1990) Tissue samples were collected using tweezers and placed immediately in liquid nitrogen. Total RNA was extracted with the RNeasy Mini Kit (Qiagen) following the manufacturer's instructions manual. RNA was treated with TURBO DNase (Invitrogen) and retro-transcribed using Superscript III and Oligo(dT)12–18 Primer (Invitrogen).

### qPCR optimization and conditions

Oligonucleotides for expression analysis of *STK* (AT4G09960), *AGO9* (AT5G21150), *SPL* (AT4G27330), *RDR6* (AT3G49500), *DRM1* (AT5G15380) and *DRM2* (AT5G14620) were designed *de novo* (Table S3). Primer specificity tests and qPCR experiments were performed as described by Burton et al. (2004) with the following qPCR cycling conditions: 3 min at 95°C, followed by 45 cycles of 10 s at 95°C, 1 s at 55°C, 30 s at 72°C and 15 s at the optimal acquisition temperature (see Table S3). The transcript levels of genes encoding AtCyclophilin (AT2G36130), AtActin (AT5G43500), AtTublin (AT1G50010) and AtGAPdH (AT3G26650) were used as controls. Normalization was carried out using these control genes as described by Burton et al. (2004). The results are expressed as arbitrary units that represent the number of copies per microlitre of cDNA, normalized against the geometric means of the three control genes that vary the least with respect to each other (Vandesompele et al., 2002). The standard error was calculated from the average expression level of each biological replicate. For the statistical analysis, a two-tailed, unpaired, homogeneity of variance Student's *t*-test was performed, with the level of significance set as *P*=0.05. Three biological replicates were analysed for the whole inflorescence experiment. At least nine technical replicates were analysed in each experiment.

### Microscopic analysis: DIC and confocal microscopy

To analyse cleared tissue, DIC microscopy (Zeiss Axiophot D1 immersion ×10, ×20, ×40 and ×100) was used. This is an optical microscopy illumination technique used to enhance the contrast in unstained and transparent samples. In particular, DIC microscopy was used to observe the percentage of single MMCs and multiple MMCs in finger-like ovules in mutant lines and the wild type. The statistical significance was analysed using Student's unpaired *t*-test. Pictures were acquired with a Zeiss Axiocam MRc5 camera and Axiovision (version 4.1) software.

To analyse premeiotic ovules in even more detail, a modification of the protocol described by Braselton et al. (1996) for confocal laser scanning microscopy was used. This treatment stains both nuclei and cell walls. Staining solution, containing 0.1% (v/v) SR2200 (Renaissance Chemicals; stock solution from the supplier was considered as 100%) in water, was prepared freshly before use. Ovules were manually dissected out of the pistil and collected in a drop of staining solution on a microscope slide and mounted under a coverslip. Images were obtained with Nikon A1 confocal microscopes. SR2200 was excited with a 405 nm laser line and emission recorded between 415 and 476 nm (405 nm/415-476 nm), similar to 4′,6-diamidino-2-phenylindole (DAPI) settings. The different mutant phenotypes were analysed by three students in two different laboratories.

### Callose staining and microscopy

Analyses of callose produced before meiosis were performed using Aniline Blue staining. Whole flowers were collected at stages corresponding to ovule stage 2-I (Schneitz et al., 1995) and dissected under a Zeiss dissection microscope. Samples were collected from the wild type and at least three confirmed homozygous lines for each mutant. Carpels were gently sliced open in 20 μl of Aniline Blue staining solution [0.005% (w/v) Aniline Blue diammonium salt (Sigma Aldrich, catalogue no. 415049) in PBS] according to the protocols of Rodkiewicz (1970) and Reiser and Fischer (1993). The ovules were gently detached from the placenta and released into the solution. A further 20 μl of Aniline Blue solution was pipetted onto the sample, immediately covered with a coverslip and transferred to a fluorescence Axio Imager M2 microscope for viewing under ultraviolet light (CFP filter; Zeiss Filter set 47: 436 nm/480 nm). Autofluorescence was used to highlight the ovule outline in the dsRED channel (Zeiss Filter set 43: 545 nm/605 nm), and the DIC images were captured with Nomarksi optics. The YFP signal (for pLC2:nlsYFP and pKNU:nlsYFP) was detected using a YFP filter (Zeiss Filter set 46: 500 nm/535 nm). Images were processed with ZEN imaging software and Adobe Photoshop.

Staining of callose during meiosis was performed with staining solution, containing 0.1% (v/v) SR2200 (Renaissance Chemicals; stock solution from the supplier was considered as 100%) in water, prepared freshly before use. For imaging, the ovules were manually dissected out of the pistil and collected in a drop of staining solution on a microscope slide and mounted under a coverslip. Images were obtained with Nikon A1 confocal microscopes. SR2200 was excited with a 405 nm laser line and emission recorded between 415 and 476 nm (405 nm/415-476 nm), similar to DAPI settings.

### Chromatin immunoprecipitation studies

ChIP assays were performed as described by Gregis et al. (2009) using for STK_GFP the commercial antibody GFP:Living Colors full-length (Clontech). qRT-PCR assays were performed to determine the enrichment of the fragments. The detection was performed in triplicate using the iQ SYBR Green Supermix (Bio-Rad) and the Bio-Rad iCycler iQ Optical System (software version 3.0a), with the primers listed in Table S4. ChIP qRT-PCR experiments and relative enrichments were calculated as reported by Matias-Hernandez et al. (2010). We used the following formulae to calculate the fold enrichment: dCT.tg=CT.i−CT.tg and dCT.nc=CT.i−CT.nc, where Ct.tg is the target gene mean value, Ct.i is the input DNA mean value, and Ct.nc is the ACTIN 7 (negative control) mean value. The propagated error values of these cycle thresholds (CTs) are calculated using dSD.tg=sqrt((SD.i)^2^+(SD.tg^2^)/sqrt(*n*) and dSD.nc=sqrt((SD.i)^2^+(SD.nc^2^)/sqrt(*n*), where *n*=number of replicate per sample. Fold-change over negative control was calculated, finding the ΔΔCT (ddCT) of the target region as follows: ddCT=dCT.tg−dCT.nc and ddSD=sqrt(dSD.tg)^2^+(dSD.nc)^2^. The transformation to linear fold-change (FC) values is obtained as follows: FC=2^(ddCT)^ and FC.error=ln(2)×ddSD×FC. All the experiments were performed in three biological replicates.

### Cloning *SPL5′::GUS:3′* and *SPL5′::SPL_GFP:3′*

Initially, the *SPL* locus was cloned into pDONR207 (Life Technologies) and subsequently transferred to the pBGWFS7 destination vector (ThermoFisher Scientific); the expression vector was used to amplify the *SPL* genomic region fused to the *GFP* reporter gene. The fragment obtained was cloned into pDONR207 (Life Technologies). The putative promoter region of the gene plus the 5′ UTR and the 3′ UTR were cloned and subsequently transferred to pDONR201 P4-P1r and pDONR221 P2r-P3, respectively. By a multisite gateway approach, we obtained *pSPL:SPL_GFP-3′UTR*, combining the obtained donor vectors (described above) and transferring them into the pH7M34GW destination vector (ThermoFisher Scientific). The primers used are listed in Table S5.

### Cloning 35S *CaMV::SPL/NZZ*

To construct *35S::SPL*, SPL CDS was amplified with the primers listed in Table S5 and cloned into the Gateway destination vector, pB2GW7. All constructs were verified by sequencing and used to transform wild-type plants using the floral dip method (Clough and Bent, 1998).

## Supplementary Material

Supplementary information

Reviewer comments
