# Supplementary material for: The RNA-dependent DNA methylation pathway is required to restrict SPOROCYTELESS/NOZZLE expression to specify a single female germ cell precursor in Arabidopsis
Source: Development. 2020 Dec 13;147(23):dev194274. doi: 10.1242/dev.194274 (PMC7758631; doi:10.1242/dev.194274)
Supplement: Supplementary information [file develop-147-194274-s1.pdf]

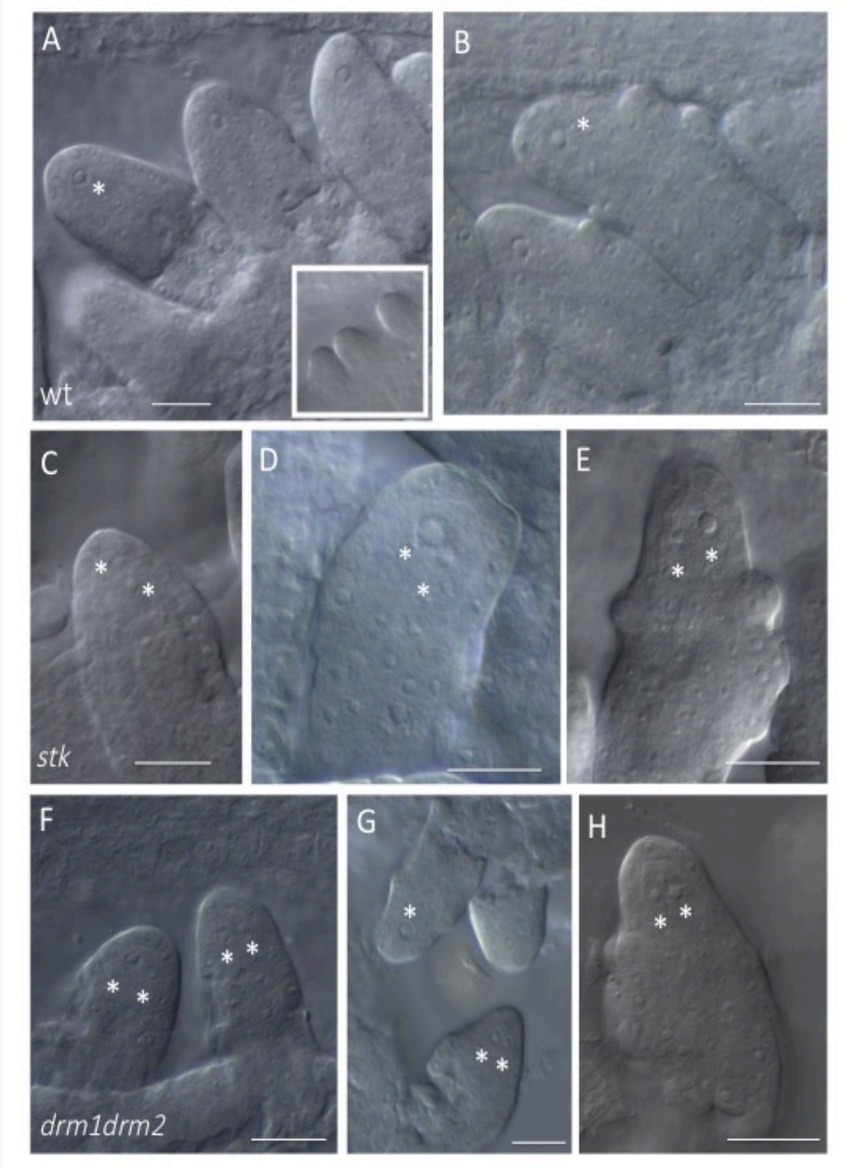

Figure S1. DIC analysis of ovule primordium. (A-B) wild-type, during the first stages of ovule primordia development any enlarged cell are detected - small detail in A, only after the ovule elongation, at finger like stage, a single enlarged cell is detectable corresponding to the MMC is detectable; (C-F) *stk* mutant; (G-I) *drm1drm2* double mutant. Multiple enlarged cells - MMC-like cells - were detected in several mutant ovules nucellus and are marked with a white asterisks.

Development • Supplementary information

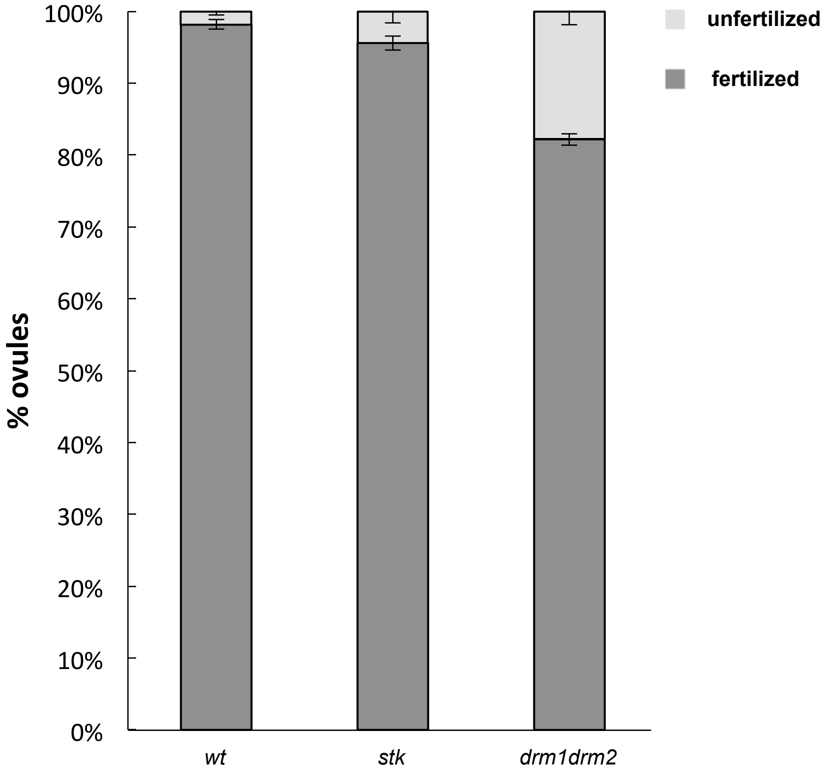

Figure S2. Fertility analyses in the selected mutants. The number of fertilized and unfertilized ovules were counted in *stk* mutant and *drm1drm2* double mutant in comparison with wild-type.

Development • Supplementary information

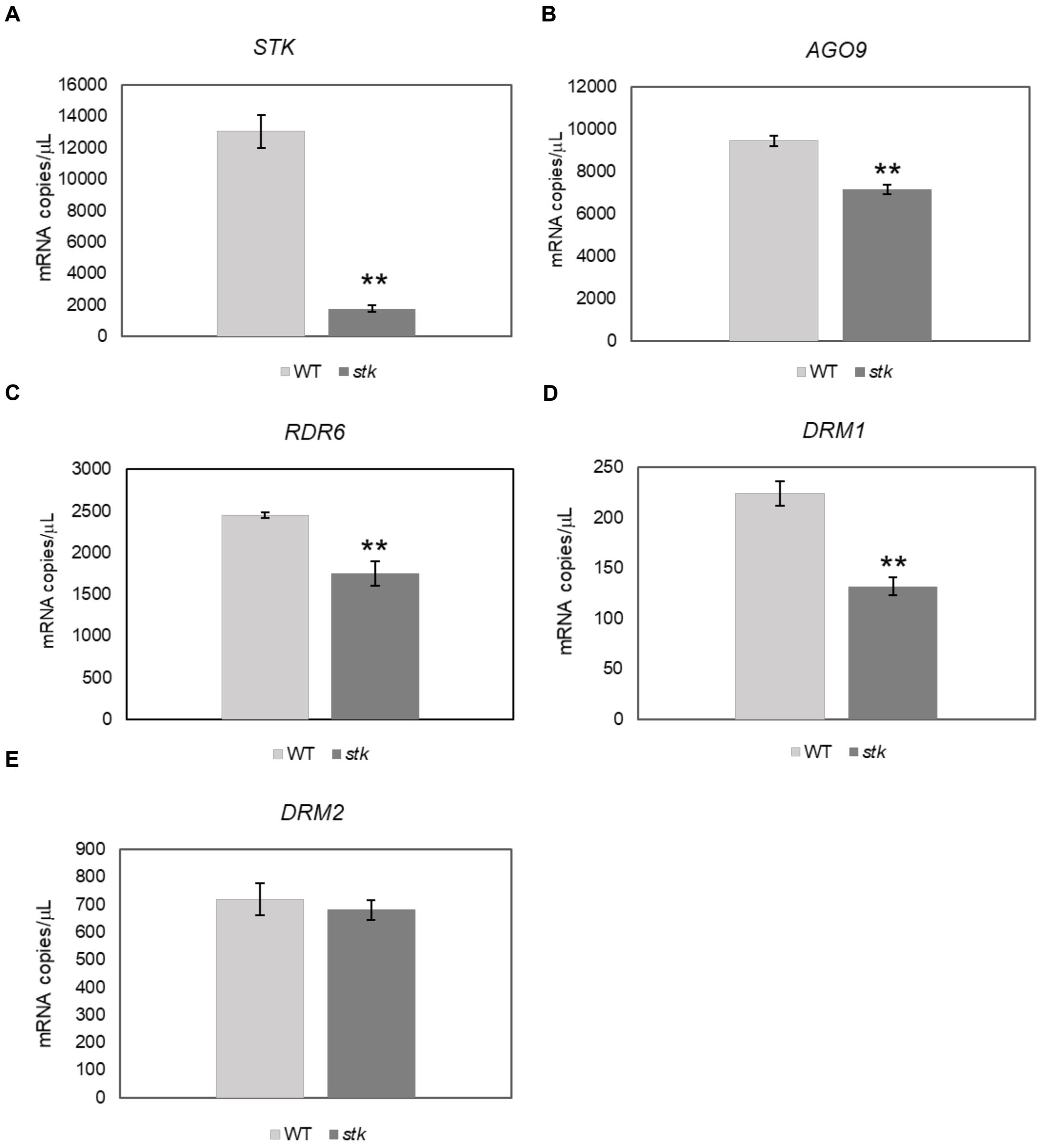

Figure S3. Gene expression analysis in wt and *stk* inflorescences and flowers. (A-E) Quantitative PCR was used to examine expression of *STK* , *AGO9*, *RDR6*, *DRM1* and *DRM2* in whole inflorescences. Asterisks (**) indicate significant differences in *stk* compared to wt and represent a *p*-value < 0.01 in a Student's t-test.

Development • Supplementary information

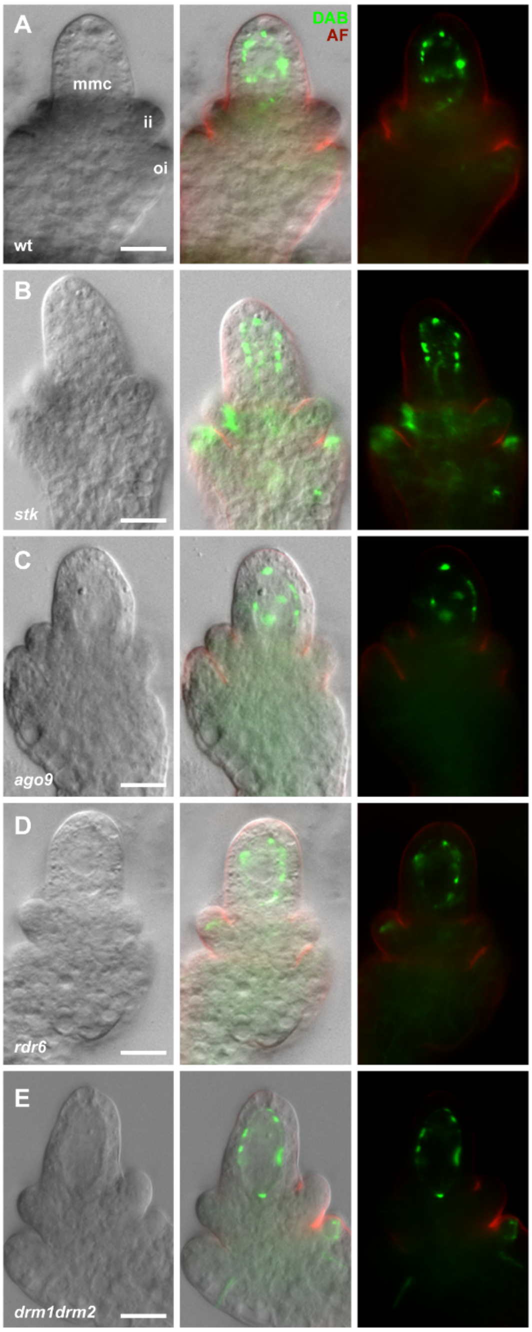

Figure S4. Callose accumulation during megaspore mother cell expansion.
Ovule from wt (A), *stk* (B), *ago9* (C) *rdr6* (D) and *drm1drm2* (E) mutant backgrounds were stained with decolourised aniline blue (DAB) and examined at stage 2-I (MMC stage); representative ovules are shown. Callose staining is indicated in green, while autofluorescence (AF) is indicated in red; ii, inner integument; mmc, megaspore mother cell; oi, outer integument. Scale bar = 12µm.

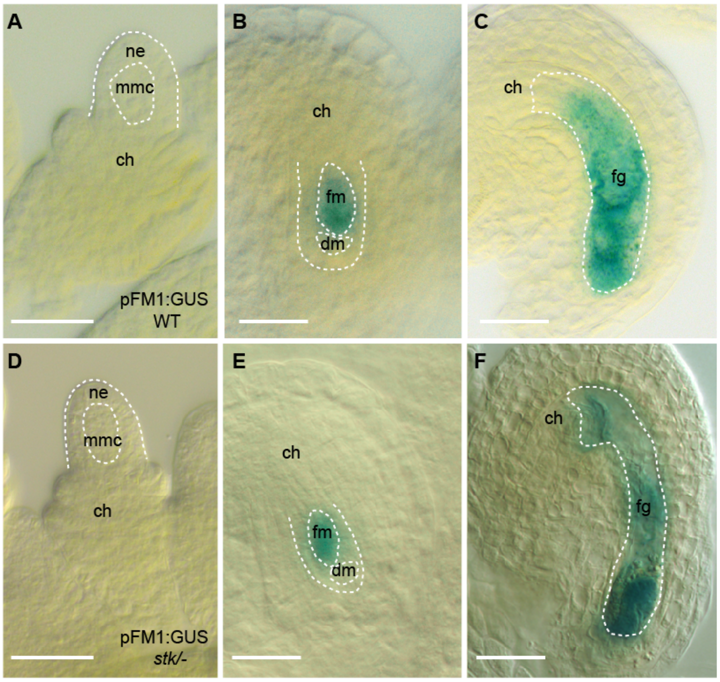

Figure S5. *pFM1::GUS* expression analysis. (A-C) In wild-type the marker is absent at the MMC stage an it is only detectable at the functional megaspore (fm) and in the developing female gametophyte (fg). (D-F) In *stk* mutant any differences were detected.

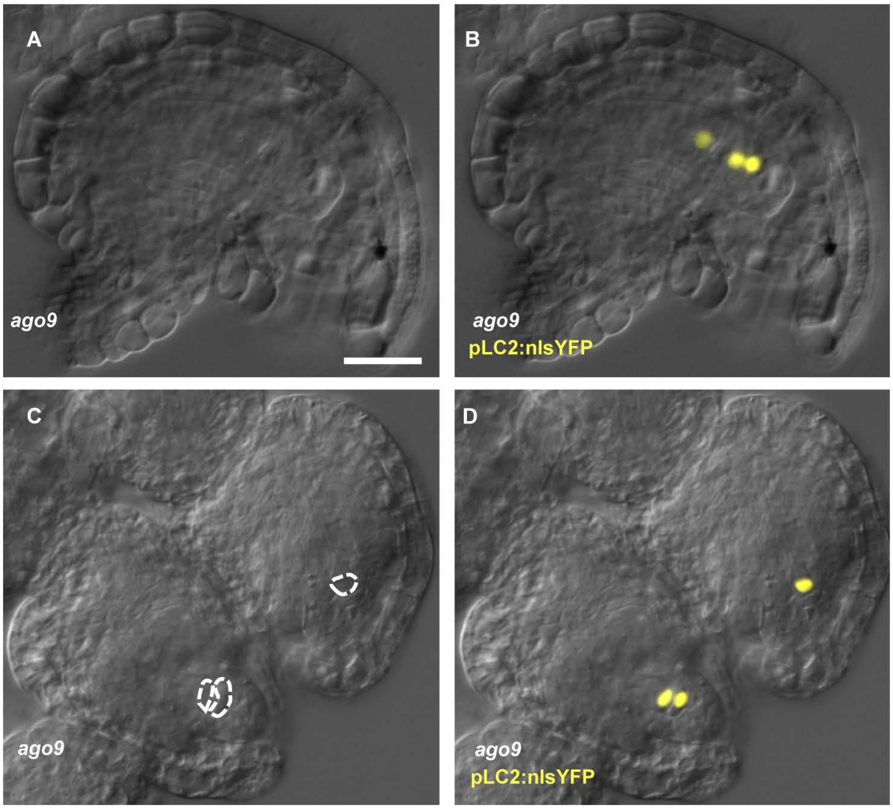

Figure S6. *pLC2:nlsYFP* expression in *ago9-2* mutant.
pLC2:nlsYFP marker in wild-type (A) and *ago9-2* single mutant (B). Inactivation of AGO9, leads to subtle changes in *pLC2:nlsYFP* expression in less than 2% (9/440) of the analyzed ovules. Two signal sources were detected; however, it was unclear whether this signal was in one functional megaspore and one supernumerary enlarged cell, or two megaspores within the meiotic tetrad.

Development • Supplementary information

A

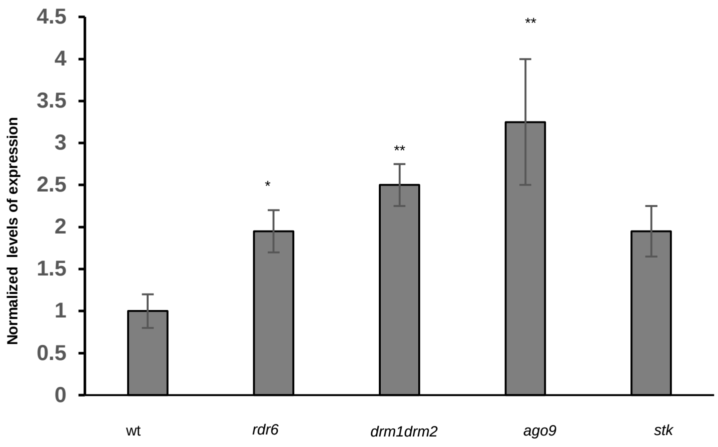

B

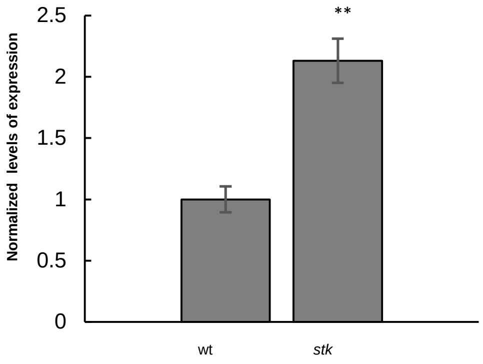

Figure S7. **qRT-PCR analysis of *SPL/NZZ* expression.** (A) Expression analysis of SPL/NZZ by qRT-PCR in wild-type, *rdr6, drm1drm2, ago9 and stk* closed flowers. The expression of *SPL* was normalized to that of ubiquitin and the expression level in wild-type was set to 1. Error bars represent the standard error mean of three biological replicates; analysis of variance (ANOVA) and post-hoc Tukey honestly significant difference (HSD) test were used; Asterisks (*) and (**) indicate significant differences in the mutants background compared to the wild-type and represent a p-value < 0.05 and p < 0.01, respectively. (B) Expression analysis of SPL/NZZ by qRT-PCR in wild-type and stk mutants in selected stages, encompassing megasporogenesis. Asterisks (**) indicate significant differences in the mutant background compared to the wild-type and represent a p-value < 0.0

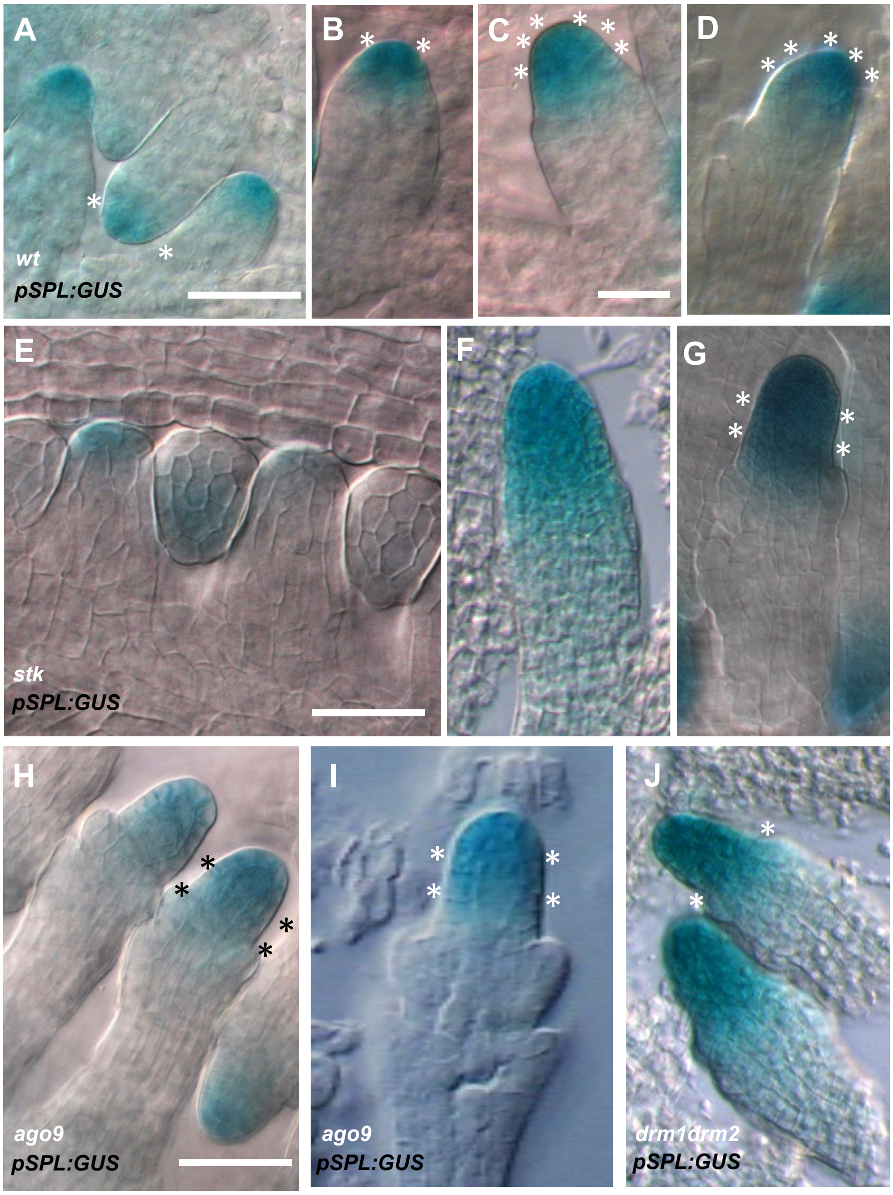

Figure S8. Mutation in STK and in the rdDM pathway affects pSPL expression domain. Analysis of *pNZZ/SPL5'::GUS:3 '* expression in wt (A-D); *stk (*E-G), *drm1drm2, ago9-2* (H-I) and *drm1drm2 (J)* mutants background; scale bar= 15 µm. Asteriks highlight the specific expression domain of pSPL, confined to L1 nucella; in the mutant backgrounds analysed we could observe an extention of the domain, no longer restricted to tip of the nucellus.

Development • Supplementary information

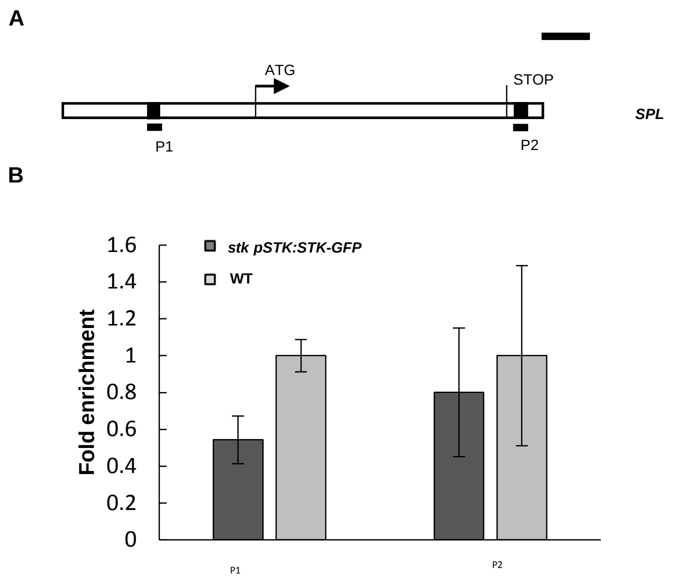

Figure S9. STK do not directly bind to the *SPL* locus. (A) Schematic diagram of the *SPL* locus, indicating the regions analysed by chromatin immunoprecipitation (ChIP; black bars); black boxes, CArG boxes; scale bar =500 bp. (B); Quantitative Real-Time PCR analysis of ChIP assay using chromatin extracted from *pSTK::STK_GFP* and wt (as a negative control), testing the CArG boxes regions on *SPL* locus. For the IP, commercial antibodies against GFP were used; Error bars represent the propagated error value using three replicates. ChIP results of one representative experiment are shown. Positive binding site fragments were considered only if they were enriched compared with the controls in at least three independent experiments.

Development • Supplementary information

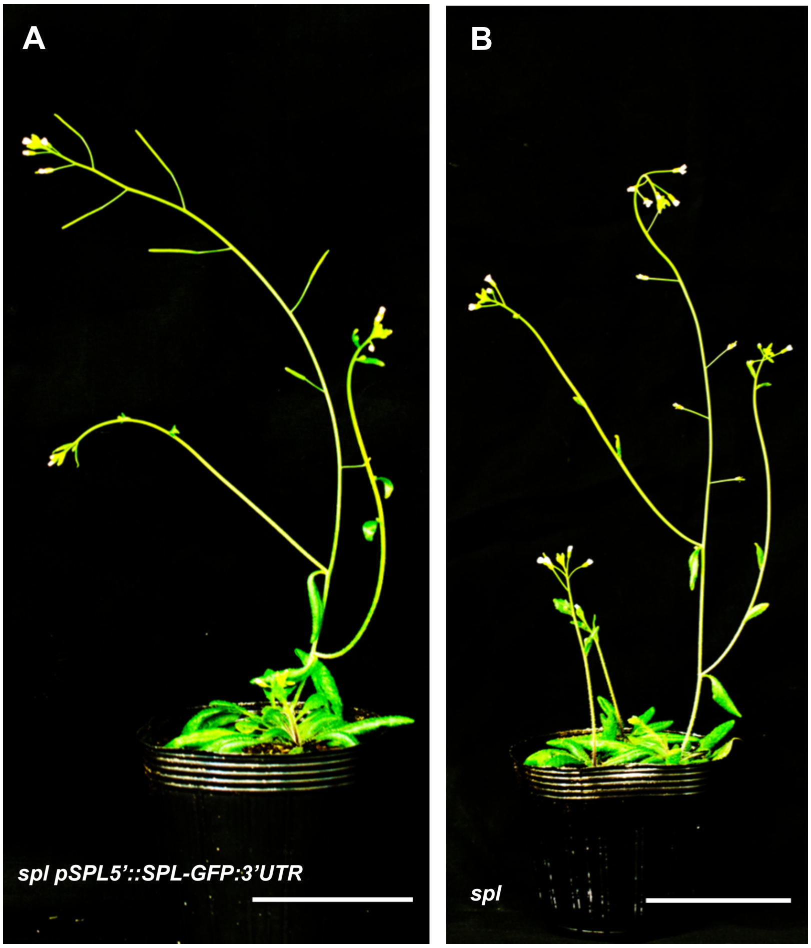

Figure S10. **Complementation analysis**. (A) *spl/nzz* mutant complemented with *pSPL5'UTR::SPL-GFP:3'UTR*; (B) *spl* mutant; scale bar=5 cm.

Development • Supplementary information

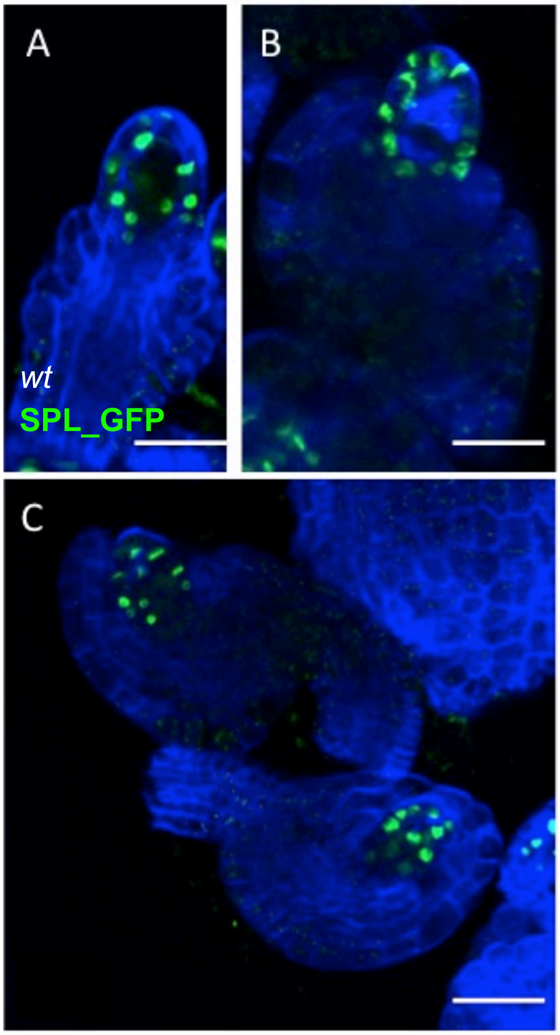

Figure S11. *SPL_GPF* expression analysis during meiosis. Analysis of SPL/NZZ protein localization using *pSPL_5'UTR::SPL-GFP_3'UTR* .(A-C) In wild-type SPL expression during meiosis is restricted to the layers that involve the MMC that goes in meiosis. In blue Renaissance staining.

Development • Supplementary information

Table S1. *pKNU::nlsYFP* analysis in all mutant backgrounds

**Mutant**	**Single cell**	**Two cells with expression**	**No expression (or v. weak)**	**Total**
stk	290	0	178	468
	**62%**	**0%**	**38%**	
SD	3%		2%	
wt	411	0	51	462
	**89%**	**0%**	**11%**	
SD	2%		2%	
drm1drm2	349	0	222	571
	**61%**	**0%**	**39%**	
SD	3%		2%	
wt	142	0	11	152
	**93%**	**0%**	**7%**	
SD	3%		2%	
ago9-2	623	12	48	683
	**91%**	**2%**	**7%**	
SD	3%	0.5%	2%	
wt	659	15	14	688
	**96%**	**2%**	**2%**	
SD	1%	0.5%	2%	
rdr6-11	280	0	70	350
	**80%**	**0%**	**20%**	
**SD**	3%		3%	
wt	99	0	11	110
	**90%**	**0%**	**10%**	
**SD**	1%		2%	

Development • Supplementary information

Table S2. Mutant and marker lines of *Arabidopsis* used in this study

Mutant	AT*G	Allele	Line
drm1drm2	AT5G15380 (*DRM1*)	*drm1-2*	SALK_021316
	AT5G14620 (*DRM2*)	*drm2-2*	SALK_150863
ago9-2	AT5G21150	*ago9-2*	SALK_112059
spl	AT4G27330	*spl-1*	T-DNA
stk	AT4G09960	*stk-2*	TE
rdr6-11	AT3G49500	*rdr6-11*	N24285

Marker line	Publication
pKNU::YFP	Tucker et al., 2012
pLC2::YFP	Tucker et al., 2012

Table S3. Primers used for qPCR experiments

Gene name	Gene	Sequence	Amplicon size	Acquisition temperature
AGO9	AT5G21150	CACCTTTCGTTCCAGCAAAT<br>CCGCTTGGTTTGTTGAACTT	154	79°C
RDR6	AT3G49500		187	75°C
SPL/NZZ	AT4G27330	CTTGGGAAGCCTTGTAGCAC<br>AGCTCGAGCGTCAGAGAATC	148	75°C
DRM1	AT5G15380	CCAATTCCGGATATTGCTCGTG<br>TTGGCCCAAACACCTTTTGG	80	75°C
DRM2	AT5G14620	TGTGCTGCTCAAATGGCTAG<br>GCTCTCCTTTTGACTCAAACCG	106	75°C
AtCyclophilin	AT2G36130	TGGCGAACGCTGGTCCTAATACA<br>GTCAGCCAAGTCAACAACTCTCTG	223	79°C
AtGAPdH	AT3G26650	TGGTTGATCTCGTTGTGCAGGTCTC<br>GTCAGCCAAGTCAACAACTCTCTG	262	79°C
AtActin	AT5G43500	GAGTTCTTCACGCGATACCTCCA<br>GACCACCTTTATTAACCCCATTTACCA	180	79°C
AtTubulin	AT1G50010	ATGTGGGTCAGGGTATGGAA	143	79°C

Development • Supplementary information

Table S4. Primers used in ChIP experiments

Primer name	Gene	Sequence	Amplificate size	Objective
RT_2905	AT5G21150	GTCACATCACGTTAAATCA	178	*pAGO9*
RT_2905		GTGGAAACTGTTTAACC		region 1
RT_2907	AT5G21150	AACGACGACCTGCAAAAC	300	*pAGO9*
RT_2908		TGTCATCACCTCAAATTTG		region 2
RT_2909	AT5G21150	CACAATTGTTTATTGGAACAC	137	*pAGO9*
RT_2910		CACAAAAAGAGCTATATGAAC		region 3
RT_2911	AT5G21150	GAACAACATCTTTAGCAC	220	*pAGO9*
RT_2912		CAGGTACTTAAACCGGTTATTC		region 4
RT_2293	AT3G49500	CCTTCAGTCTGTTCTCTGTTGCT	180	*pRDR6*
RT_2294		TGTGCTGCTCACGTGCTATT		region 1
RT_2295	AT3G49500	CAGTCTCCGCATTGCTTCTAT	150	*pRDR6*
RT_2296		CGATCACCTAATACATTGTTGATTG		region 2
RT_2297	AT3G49500	CAATCAACAATGTATTAGGTGATCG	110	*pRDR6*
RT_2298		CGCTACTCCGTTGGAAGATC		region 3
RT_2299	AT3G49500	GATCTTCCAACGGAGTAGCG	120	*pRDR6*
RT_2300		CCCTCTGACCCCATTTCTC		region 4
RT_0045	AT5G09810	CGTTTCGCTTTCCTTAGTGTTAGCT	134	*ACT 7*
RT_0046		AGCGAACGGATCTAGAGACTCACCTTG		
RT_0795	AT5G18000	GGGAAGGTCATGGCAAGTTA	170	*pVDD*
RT_0796		CCATCTGCCTCGAATATGGT		
RT_2915	AT4G27330	ccaagattaacatggaggca		*pSPL reg 1 fw*
RT_2916		ctcactatacttagctgac		*pSPL reg 1 rv*
RT_2917	AT4G27330	gtctgagtgtttaggttag		*SPL 3-UTR fw*
RT_2918		CCATTGATTTGTCCTTGAAGC		*SPL 3-UTR rv*

Development • Supplementary information

Table S5. Primers used for cloning

Primer name	Gene	Sequence	Amplificate size	Objective
**ATP_6475**	AT4G27330	GGGGACAAGTTTGTACAAAAAAGCAGGCTcaATGGCGACTTCTCTCTTCTTC	1171	SPL locus
**ATP_6476**	AT4G27330	GGGGACCACTTTGTACAAGAAAGCTGGGTcGAAGCTTCAAGGACAAATCAATG		SPL locus
**ATP_6548**	GFP	GGGGACCACTTTGTACAAGAAAGCTGGGTC TCACTTGTACAGCTCGTCCAT	1850	SPL-GFP
**ATP_6234**	5'_AT4G27330	GGGGACAACTTTGTATAGAAAAGTTGtagaatgcaatacatggctg	3906	5'SPL (attB4)
**ATP_6235**	5'_AT4G27330	GGGGACTGCTTTTTTGTACAAACTTGtgatgatgatcttcttctcgga		5'SPL (attB1r)
**ATP_6236**	3'_AT4G27330	GGGGACAGCTTTCTTGTACAAAGTGGatgttttatctttctatattg	1609	3'SPL (attB2r)
**ATP_6237**	3'_AT4G27330	GGGGACAACTTTGTATAATAAAGTTGgatcgtcttcttcttcttctctgc		3'SPL (attB3)
**ATP_1575**	β-glucuronidase (GUS)	CCACTGTTACGTCCTGTAGAAACCCC	1809	GUS for TOPO cloning
**ATP_1576**	β-glucuronidase (GUS)	TCATTGTTTGCCTCCCTGCTG		GUS rev
**ATP_4196**	AT4G27330	GGGGACAAGTTTGTACAAAAAAGCAGGCTTCATGGCGACTTCTCTCTTCTTC	945	SPL CDS fw
**AtP_4197**	AT4G27330	GGGGACCACTTTGTACAAGAAAGCTGGGTTTAAAGCTTCAAGGACAAATC		SPL CDS rv

Development • Supplementary information
