# Supplementary material for: The RNA-dependent DNA methylation pathway is required to restrict SPOROCYTELESS/NOZZLE expression to specify a single female germ cell precursor in Arabidopsis
Source: Development. 2020 Dec 13;147(23):dev194274. doi: 10.1242/dev.194274 (PMC7758631; doi:10.1242/dev.194274)
Supplement: Reviewer comments [file develop-147-194274_review_history.pdf]

The RNA-dependent DNA methylation pathway is required to restrict *SPOROCYTELESS/NOZZLE* expression to specify a single female germ cell precursor in *Arabidopsis*

Marta A. Mendes, Rosanna Petrella, Edoardo Vignati, Stefano Gatti, Mara Cucinotta, Sara C. Pinto, Dayton C. Bird, Veronica Gregis, Hugh Dickinson, Matthew R. Tucker and Lucia Colombo
DOI: 10.1242/dev.194274

Editor: Ykä Helariutta

Review timeline
Original submission: 19 June 2020
Editorial decision: 3 August 2020
First revision received: 25 August 2020
Editorial decision: 14 September 2020
Second revision received: 23 September 2020
Accepted: 12 October 2020

Original submission

First decision letter

MS ID#: DEVELOP/2020/194274

MS TITLE: The RNA dependent DNA methylation pathway is required to restrict SPOROCYTELESS/NOZZLE expression to specify a single female germ cell precursor in Arabidopsis

AUTHORS: Marta A. Mendes, Rosanna Petrella, Edoardo Vignati, Stefano Gatti, Mara Cucinotta, Sara C. Pinto, Dayton C. Bird, Veronica Gregis, Hugh Dickinson, Matthew R. Tucker, and Lucia Colombo

I have now received all the referees' reports on the above manuscript, and have reached a decision. The referees' comments are appended below, or you can access them online: please go to BenchPress and click on the 'Manuscripts with Decisions' queue in the Author Area.

As you will see, the referees express considerable interest in your work, but have some significant criticisms and recommend a substantial revision of your manuscript before we can consider publication. If you are able to revise the manuscript along the lines suggested, which may involve further experiments, I will be happy receive a revised version of the manuscript. Your revised paper will be re-reviewed by one or more of the original referees, and acceptance of your manuscript will depend on your addressing satisfactorily the reviewers' major concerns. Please also note that Development will normally permit only one round of major revision.

We are aware that you may currently be unable to access the lab to undertake experimental revisions. If it would be helpful, we encourage you to contact us to discuss your revision in greater detail. Please send us a point-by-point response indicating where you are able to address concerns raised (either experimentally or by changes to the text) and where you will not be able to do so within the normal timeframe of a revision. We will then provide further guidance. Please also note that we are happy to extend revision timeframes as necessary.

Please attend to all of the reviewers' comments and ensure that you clearly highlight all changes made in the revised manuscript. Please avoid using 'Tracked changes' in Word files as these are lost

© 2020. Published by The Company of Biologists under the terms of the Creative Commons Attribution License (https://creativecommons.org/licenses/by/4.0/).

in PDF conversion. I should be grateful if you would also provide a point-by-point response detailing how you have dealt with the points raised by the reviewers in the 'Response to Reviewers' box. If you do not agree with any of their criticisms or suggestions please explain clearly why this is so.

Reviewer 1

Advance summary and potential significance to field

Mendes and colleagues present a pathway for early MMC specification that links the ovule identity gene STK to RNA-Directed DNA Methylation (RdDM) to the sporocyteless gene (SPL/NZZ). RdDM mutants have previously been shown to produce ectopic MMCs; what is added here is showing that the drm1/drm2 double mutant has a similar phenotype (which I believe is a more specific defect in the RdDM pathway compared to mutants that were previously analyzed), and, importantly connecting RdDM to SPL/NZZ and STK. The model presented by the authors provides new links in a pathway that controls the number of MMCs in the ovule; unfortunately, many core conclusions in the paper are currently unconvincing, as outlined below.

Comments for the author

Major concerns:

(1) The authors define cells as putative MMCs based solely on a large cell size. However, cell size is hardly a specific criterion for cell identity. The putative “ectopic MMCs” do not show any other evidence of MMC differentiation: they are not surrounded by callus, do not express the markers pKNU:nlsYFP or pLC2:nlsYFP, and do not develop into functional gametophytes. Unfortunately with ectopic MMCs being defined solely by size, it was not convincing that any of the mutants actually produced extra MMCs. Almost every claim in the paper is dependent on this definition of “ectopic MMC”.
In Olmedo-Monfil et al. 2010, the ago9-2 mutant was shown to express ectopic pFM2:GUS, a megaspore marker. Why was this marker not tested to see if the authors can reproduce these prior results?

(2) The absence of SPL/NZZ transcript / protein in MMCs seems contrary to previous reports. For instance, in Yang, et al. (1999) they showed that SPL transcript and promoter activity is strong in the MMCs (in addition to neighboring cells). Why do the authors feel their results were different? A better discussion of the discordance between the results of this paper and prior evidence of SPL/NZZ expression is warranted. Claims made based on the absence of SPL/NZZ expression in MMCs should also be toned down unless a convincing rationale for the disagreement with prior data is given.

There are a few areas where the statistics were not calculated or where the analysis needs to be more clearly described:

-Fig. 2B,C; Fig. S6 – Tests for statistical significance should be performed for the ChIP experiments. Since the conclusions would change if any site is deemed significant (e.g. a ChIP enrichment at any of P1-4 in Fig 2B would be interpreted as evidence of binding), the statistical test should absolutely be adjusted to account for multiple hypothesis testing.

-Fig. S5 – For the qRT-PCR results, many comparisons are possible and it is not clear which were made. Was expression in each of the mutants individually compared to the wild type control? A Tukey’s Honest Significance Difference Test may be most appropriate for this analysis and would account for multiple hypothesis testing.

-qRT-PCR analysis (Method section) – Samples were obtained with 3 technical replicates each for 3 biological replicates. It was not stated how the technical replicates were handled when quantifying the expression changes or assessing statistical significance. Was each technical replicate treated as an independent sample? Or were the technical replicates averaged and only the biological replicates treated as independent? (the second is the correct analysis)

© 2020. Published by The Company of Biologists under the terms of the Creative Commons Attribution License (https://creativecommons.org/licenses/by/4.0/).

Other comments:

-It is not accurate to refer to the MMC as the “female germline”. The MMC is the precursor for antipodal cells, synergid cells, etc. in addition to the egg cell, and so the germ lineage is not yet specified until later in gametophyte development.

-The manuscript would be strengthened if the microscopy quantification of large cells were performed blind (e.g. Fig 1C, 1H). Given how subtle the extra enlarged cells are to my eye, I might expect even the most honest and careful scientist to unintentionally exaggerate the difference between wild type and mutant if the sample identity were known. If the images were to be reanalyzed at this point, it would be recommended to mask the sample identities and then have someone score the images who has not looked at them before.

-Claims that SPL/NZZ acts “non-cell autonomously” were confusing, as this statement suggests that SPL has a biochemical function in a cell different from the site of synthesis – which is not what the authors show. The statement “SPL/NZZ acts non-cell autonomously” is not equivalent to the statement “SPL/NZZ triggers a pathway that acts non-cell autonomously”. The writing could be clarified by re-examining claims of cell autonomy and making sure they say what the authors intend.

Reviewer 2

Advance summary and potential significance to field

The manuscript by Mendes et al. is interesting. It demonstrates that several key players of the RdDM pathway are important for restricting SPL/NZZ to the tip of the ovule primordium. They also provide ChIP data using a STK-GFP fusion and searched for CArG boxes in the promoter of either RDRD6, AGO9, DRM1 and DRM2. They identified significant enrichment for different regions in the promoter of either RDR6 of AGO9, but failed to identify similar pattern fr DRM1 and DRM2. They also show that pKNU::nlsYFP and/or pLC2::nlsYFP do not indicate specific alterations in the mutants, and they conclude that most likely, neither of the mutants undergoes a commitment to meiosis, or funcional megaspore identity. The paper is well documented, is scientifically sound, and provides important information on the potential regulation of SEEDSTCIK (STK) via the RdDM pathway, and directly or indirectly SPL/NZZ.

Comments for the author

One issue that I have with the paper is the overly optimistic number of cells that they document on their graphs. In my experience, most RdDM components only express 15 to 25% of additional MMCs, while here the authors report up to 65% in drm12, and 46% in stk. While they used the Olmedo-Monfil et al paper in Nature as a benchmark, it is unclear whether the « extra » MMCs are indeed MMCs. A conspicious aspect of MMC identity is the presence of an enlarged nucleolus, but in the images that I looked at, I cannot really identify much differences between the somatic nucleuli (by Feulgen) and the extra MMCs (Fig1 A-B, versus Fig1 D-E, and Fig1 F-G). Also, the images are clearly at different timing of ovule development, particularly the stk mutant (is it stage 1-II, 2-I ?). The data for the 35S looks more convincing, as it clearly shows proper enlarged nucleoli.

A really interesting aspect of the paper is the upregulation of most of the RdDM mutants and stk in SPL/NZZ. Together with the pSPL fusion, this suggests that indeed SPL/NZZ acts non-cell autonomously. This is an important results, which credibly shows that RdDM and STK are essential both genetically an epigenetically for MMC differentiation.

Overall, the work is well documented, but I would suggest that authors might consider looking at ovules at earlier stages of ovule development.

© 2020. Published by The Company of Biologists under the terms of the Creative Commons Attribution License (https://creativecommons.org/licenses/by/4.0/).

First revision

Author response to reviewers' comments

Reviewer 1 Advance Summary and Potential Significance to Field:

Mendes and colleagues present a pathway for early MMC specification that links the ovule identity gene STK to RNA- Directed DNA Methylation (RdDM) to the sporocyteless gene (SPL/NZZ). RdDM mutants have previously been shown to produce ectopic MMCs; what is added here is showing that the drm1/drm2 double mutant has a similar phenotype (which I believe is a more specific defect in the RdDM pathway compared to mutants that were previously analyzed), and, importantly, connecting RdDM to SPL/NZZ and STK. The model presented by the authors provides new links in a pathway that controls the number of MMCs in the ovule; unfortunately, many core conclusions in the paper are currently unconvincing, as outlined below.

Reviewer 1 Comments for the Author:
Major concerns:

(1) The authors define cells as putative MMCs based solely on a large cell size. However, cell size is hardly a specific criterion for cell identity. The putative "ectopic MMCs" do not show any other evidence of MMC differentiation: they are not surrounded by callus, do not express the markers pKNU:nlsYFP or pLC2:nlsYFP, and do not develop into functional gametophytes. Unfortunately, with ectopic MMCs being defined solely by size, it was not convincing that any of the mutants actually produced extra MMCs. Almost every claim in the paper is dependent on this definition of "ectopic MMC".
In Olmedo-Monfil et al. 2010, the ago9-2 mutant was shown to express ectopic pFM2:GUS, a megaspore marker. Why was this marker not tested to see if the authors can reproduce these prior results?
-We completely agree with the reviewer the extra-enlarged cells that we clearly found in the nucellus of the mutant's ovule primordium are clearly not ectopic MMC's, as they do not present any marker and do not enter in meiosis. In the very beginning of the MS we address these extra-enlarged cells as "multiple MMC-like cells" and after the marker analysis we switch this to use "multiple germline precursors" as we discuss that these extra cells do not acquire specification of MMC. This terminology is a controversial issue in the field – recently we attempted to address it in a review article (Pinto et al., Trends in Plant Science, 2019). To our mind the use of the term "germline precursor" is appropriate and it does offer some clarity to non-expert readers. If you prefer, we are happy to refer to them as multiple enlarged hypodermal cells and/or enlarged companion cells.

-Regarding the gametophytic marker present by Olmedo-Monfil et al. in 2010 *pFM2::GUS*, we were never able to germinate the seeds from this marker in the lab. However, we were able to grow and cross *pFM1::GUS* with *stk* mutant (also published by Olmedo-Monfil et al., in 2010 to be ectopically expressed in both cells in *ago9* mutants). We did not detect any obvious difference when compared to wild-type situation. However, we also find it very hard to analyse GUS reporters during these specific stages of ovule development as GUS quickly spreads to the neighbour cells; the analysis of *pLC2::YFP* with a nuclear localization signal is very much precise and solid. We have added in the supp. material a new figure with the results of *pFM1::GUS*.

Towards the end of the MS we show that the presence of this extra enlarged cells is linked to the ectopic expression of *pSPL::GUS*, and also *SPL_GFP* expression in ovules that show more than one enlarged cell.

© 2020. Published by The Company of Biologists under the terms of the Creative Commons Attribution License (https://creativecommons.org/licenses/by/4.0/).

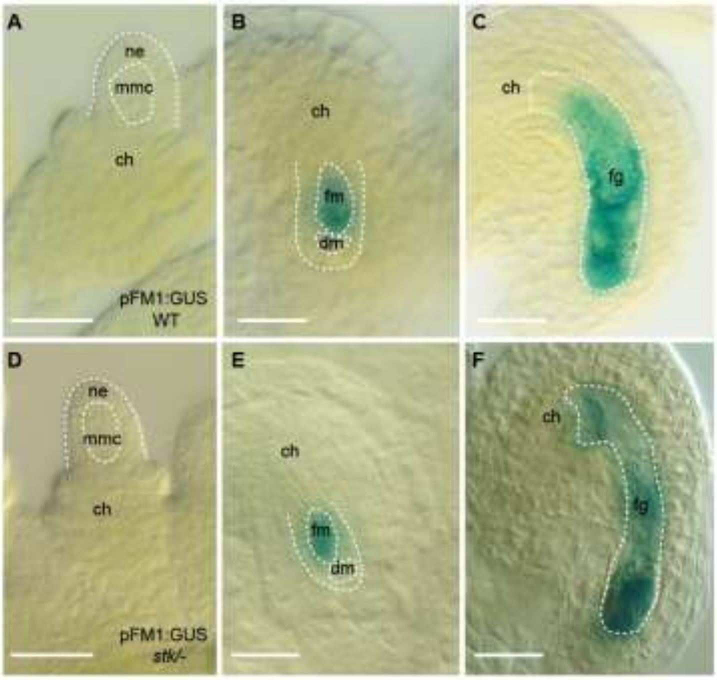

Supplemental figure 5.

(2) The absence of SPL/NZZ transcript / protein in MMCs seems contrary to previous reports. For instance, in Yang, et al. (1999) they showed that SPL transcript and promoter activity is strong in the MMCs (in addition to neighboring cells). Why do the authors feel their results were different? A better discussion of the discordance between the results of this paper and prior evidence of SPL/NZZ expression is warranted. Claims made based on the absence of SPL/NZZ expression in MMCs should also be toned down unless a convincing rationale for the disagreement with prior data is given.

-We agree with the reviewer that this observation is unexpected and contradicts much of the previous literature. For this reason, we have been very careful in our experiments; for example, we have shown the absence of *SPL/NZZ* expression in MMCs using *SPL_GFP*, a construct that unambiguously functionally complements the *spl* mutant phenotype.

As this reviewer points out, the submitted version of the MS the images relating to this part of the work may not have been fully convincing. We have therefore carried out confocal analysis of these lines using Renaissance staining. This staining marks the cell walls well, and the disposition of nuclear signal makes it far clearer to determine whether the construct is expressed inside or outside the MMC. Figure 5 now clearly shows the fusion protein to be localized in the L1 layer of the wild type ovule primordia. Moreover, as renaissance staining perfectly marks the plates formed during meiosis we further analysed the *SPL_GFP* fusion protein expression during meiosis, where again fusion protein was in the surrounding cells (please see supplementary figure 8).

Importantly, a recent report from the Xumei Cheng group in Plant Cell (Su et al., 2020) supports our finding that the expression of SPL promoter is confined to the L1 layer.

New *SPL_GFP* figure 5.

© 2020. Published by The Company of Biologists under the terms of the Creative Commons Attribution License (https://creativecommons.org/licenses/by/4.0/).

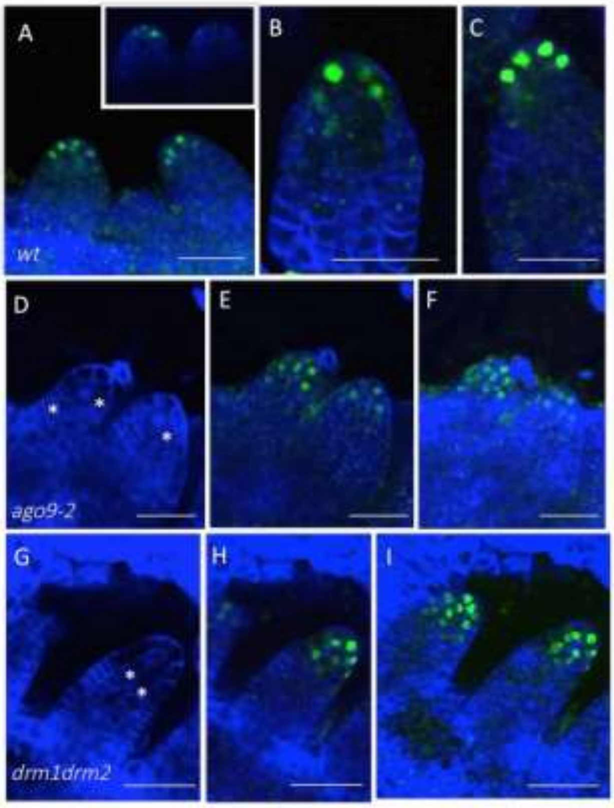

Supplementary figure 11: *SPL_GFP* during meiosis

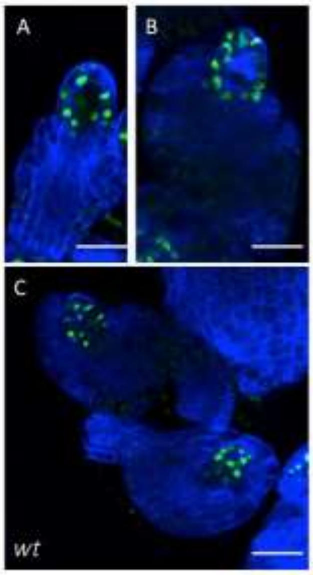

There are a few areas where the statistics were not calculated or where the analysis needs

© 2020. Published by The Company of Biologists under the terms of the Creative Commons Attribution License (https://creativecommons.org/licenses/by/4.0/).

to be more clearly described:

-Fig. 2B,C; Fig. S6 - Tests for statistical significance should be performed for the ChIP experiments. Since the conclusions would change if any site is deemed significant (e.g. a ChIP enrichment at any of P1-4 in Fig 2B would be interpreted as evidence of binding), the statistical test should absolutely be adjusted to account for multiple hypothesis testing.

As suggested by the reviewer, these data were statistically tested using the Student's t-test which showed the binding of STK to RDR6 (both regions), and to AGO9 regulatory regions, to be statistically significant with a p-value <0.05. We have added this information in the Figure 2.

-Fig. S5 - For the qRT-PCR results, many comparisons are possible and it is not clear which were made. Was expression in each of the mutants individually compared to the wild type control? A Tukey's Honest Significance Difference Test may be most appropriate for this analysis and would account for multiple hypothesis testing.

-We agree with the reviewer that some of the text may have been difficult to interpret, and we apologize for this. In the MS we reported Student's t-test comparing wt and mutant background expression but, as the reviewer has suggested we have now performed Anova followed by Tukey's Honest Significance Difference. These tests confirmed SPL up-regulation in *ago9*, *rdr6* and *drm1drm2*, whereas the *SPL* up-regulation in *stk* background was not significant. We considered that this result may well have been due to the lack of sufficient true megaspore stages in the material used for RNA extraction. We therefore performed a new qPCR experiment, enriching for megasporogenesis stages. We carried out qRT-PCR on only megasporogenesis selected stages and, interestingly, detected a slight but significant up-regulation of SPL transcripts. We have updated the text and figure Figure S5 with these results. We also attach herewith the statistical test data, accounting for all different hypotheses.

treatments pair	Tukey HSD Q statistic	Tukey HSD p-value	Tukey HSD inferfence
A vs B	9.2529	0.0010053	** p<0.01
A vs C	9.3814	0.0010053	** p<0.01
A vs D	1.3783	0.8491302	insignificant
A vs E	4.6468	0.0344941	* p<0.05
B vs C	0.1285	0.8999947	insignificant
B vs D	7.8746	0.0010053	** p<0.01
B vs E	4.6061	0.0364257	* p<0.05
C vs D	8.0031	0.0010053	** p<0.01
C vs E	4.7346	0.0306535	* p<0.05
D vs E	3.2685	0.1948972	insignificant

Where: A= wild-type

B= ago9

C = drm1drm2

D =stk

E = rdr6

The statistical analysis comparing SPL expression in the wild-type versus the mutant backgrounds was added in the figure S5.

© 2020. Published by The Company of Biologists under the terms of the Creative Commons Attribution License (https://creativecommons.org/licenses/by/4.0/).

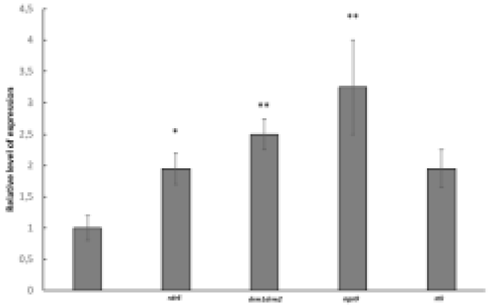

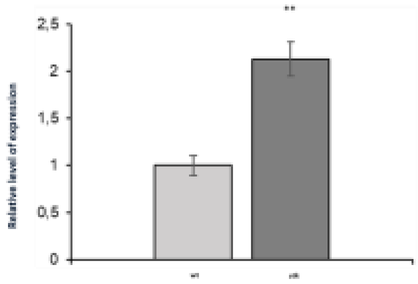

Supplemental Figure S7.

-qRT-PCR analysis (Method section) – Samples were obtained with 3 technical replicates each for 3 biological replicates. It was not stated how the technical replicates were handled when quantifying the expression changes or assessing statistical significance. Was each technical replicate treated as an independent sample? Or were the technical replicates averaged and only the biological replicates treated as independent? (the second is the correct analysis).

We apologise for the lack of clarity in the text, we performed the second (correct) analysis because technical replicates were averaged, with only the biological replicates being treated as independent.

Other comments:

-It is not accurate to refer to the MMC as the "female germline". The MMC is the precursor for antipodal cells, synergid cells, etc. in addition to the egg cell, and so the germ lineage is not yet specified until later in gametophyte development.

-We do agree that this is a controversial matter; we call it germline as in the last years many groups decided to adopt this terminology as it is easy for researchers in multiple fields to understand where the female germline starts. A recent review in Trends on Plant Science, Pinto et al 2019 summarises all the different studies where the term female germline is being used. Having said that, if the review prefers, we can change to hypodermal cells as we already suggest above.

-The manuscript would be strengthened if the microscopy quantification of large cells were performed blind (e.g. Fig 1C, 1H). Given how subtle the extra enlarged cells are to my eye, I might expect even the most honest and careful scientist to unintentionally exaggerate the difference between wild type and mutant if the sample identity were known. If the images were to be reanalyzed at this point, it would be recommended to mask the sample identities and then have someone score the images who has not looked at them before.

- We also agree that this is an excellent strategy to be used. The mutant analyses in particular of *stk* and *drm1drm2* mutants were analysed by 3 different master students without knowing which phenotype to access, in different labs across the world, and all the students reported the extra enlarged cells. These results are then documented in the numerous experiments presented in the

© 2020. Published by The Company of Biologists under the terms of the Creative Commons Attribution License (https://creativecommons.org/licenses/by/4.0/).

MS: Clearing analysis, marker crossing, meiosis staining.

-Claims that SPL/NZZ acts "non-cell autonomously" were confusing, as this statement suggests that SPL has a biochemical function in a cell different from the site of synthesis - which is not what the authors show. The statement "SPL/NZZ acts non-cell autonomously" is not equivalent to the statement "SPL/NZZ triggers a pathway that acts non- cell autonomously". The writing could be clarified by re-examining claims of cell autonomy and making sure they say what the authors intend.

- We agree with the Reviewer that this matter needs clarification. As mentioned above we performed new imaging with the *SPL_GFP* line and it is clear that the protein fusion is expressed outside the MMC in the L1 layer therefore we have corrected the text that SPL/NZZ triggers a pathway that acts non-cell autonomously.

Reviewer 2 Advance Summary and Potential Significance to Field:
The manuscript by Mendes et al. is interesting. It demonstrates that several key players of the RdDM pathway are important for restricting SPL/NZZ to the tip of the ovule primordium. They also provide ChIP data using a STK-GFP fusion and searched for CArG boxes in the promoter of either RDRD6, AGO9, DRM1 and DRM2. They identified significant enrichment for different regions in the promoter of either RDR6 of AGO9, but failed to identify similar pattern fr DRM1 and DRM2. They also show that pKNU::nlsYFP and/or pLC2::nlsYFP do not indicate specific alterations in the mutants, and they conclude that most likely, neither of the mutants undergoes a commitment to meiosis, or funcional megaspore identity. The paper is well documented, is scientifically sound, and provides important information on the potential regulation of SEEDSTCIK (STK) via the RdDM pathway, and directly or indirectly SPL/NZZ.

Reviewer 2 Comments for the Author:

One issue that I have with the paper is the overly optimistic number of cells that they document on their graphs. In my experience, most RdDM components only express 15 to 25% of additional MMCs, while here the authors report up to 65% in drm12, and 46% in stk. While they used the Olmedo-Monfil et al paper in Nature as a benchmark, it is unclear whether the extra MMCs are indeed MMCs. A conspicious aspect of MMC identity is the presence of an enlarged nucleolus, but in the images that I looked at, I cannot really identify much differences between the somatic nucleuli (by Feulgen) and the extra MMCs (Fig1 A-B, versus Fig1 D-E, and Fig1 F-G). Also, the images are clearly at different timing of ovule development, particularly the stk mutant (is it stage 1-II, 2-I ?). The data for the 35S looks more convincing, as it clearly shows proper enlarged nucleoli.

We do agree with the reviewer that the stages in Figure 1 may not be exactly the same - to address this we added a new supplementary file S11 with more images carefully-staged, cleared cells for each mutant line which we hope will clarify the multiple enlarged cell situation. We have also changed the picture for *stk* mutant in Figure 1F so that the stage is similar in all the analysed mutants.

Regarding the percentages, we have analysed a large number of ovules and indeed in our growing conditions, these are the numbers that we obtained. They are very close to those published by Olmedo Monfil in 2010 for the *ago9-2* allele of 47%. We also observe a percentage of wt ovules that present multiple enlarged cells. The mutants were analysed by 3 different master students and a post-doc in different labs so our analyses includes a great number of ovules analysed.

© 2020. Published by The Company of Biologists under the terms of the Creative Commons Attribution License (https://creativecommons.org/licenses/by/4.0/).

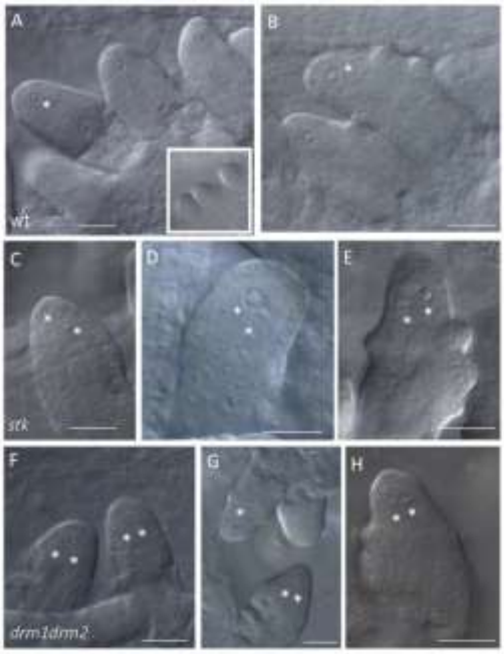

Supplementary figure 1.

A really interesting aspect of the paper is the upregulation of most of the RdDM mutants and stk in SPL/NZZ. Together with the pSPL fusion, this suggests that indeed SPL/NZZ acts non-cell autonomously. This is an important results, which credibly shows that RdDM and STK are essential both genetically an epigenetically for MMC differentiation.

-We thank the reviewer for the observation and we also added new pictures for *SPL_GFP* expression that clearly demonstrates that the *SPL/NZZ*-dependent pathway acts non cell autonomously.

Overall, the work is well documented, but I would suggest that authors might consider looking at ovules at earlier stages of ovule development.

Two figures have been added (see above)

<u>Second decision letter</u>

MS ID#: DEVELOP/2020/194274

MS TITLE: The RNA dependent DNA methylation pathway is required to restrict SPOROCYTELESS/NOZZLE expression to specify a single female germ cell precursor in Arabidopsis

AUTHORS: Marta A. Mendes, Rosanna Petrella, Edoardo Vignati, Stefano Gatti, Mara Cucinotta, Sara C. Pinto, Dayton C. Bird, Veronica Gregis, Hugh Dickinson, Matthew R. Tucker, and Lucia Colombo

I have now received all the referees reports on the above manuscript, and have reached a decision. The referees' comments are appended below, or you can access them online: please go to BenchPress and click on the 'Manuscripts with Decisions' queue in the Author Area.

The overall evaluation is positive and we would like to publish a revised manuscript in Development, provided that the referees' comments can be satisfactorily addressed. Please attend

© 2020. Published by The Company of Biologists under the terms of the Creative Commons Attribution License (https://creativecommons.org/licenses/by/4.0/).

to all of the reviewers' comments in your revised manuscript and detail them in your point-by-point response. If you do not agree with any of their criticisms or suggestions explain clearly why this is so.

We are aware that you may currently be unable to access the lab to undertake experimental revisions. If it would be helpful, we encourage you to contact us to discuss your revision in greater detail. Please send us a point-by-point response indicating where you are able to address concerns raised (either experimentally or by changes to the text) and where you will not be able to do so within the normal timeframe of a revision. We will then provide further guidance. Please also note that we are happy to extend revision timeframes as necessary.

Reviewer 1

Advance summary and potential significance to field

The revised version of this manuscript is much improved. In particular, the quantification and statistical analysis of the data is more complete and stronger; areas that were insufficiently analyzed or unclear have largely been corrected. The new imaging of the SPL-GFP fusion is a beautiful addition and made it much easier to see the expression pattern of this construct.

Currently, the key weakness in the previous version still remains.
Specifically, the core phenotype linking the drm1drm2 double mutant to hypodermal/MMC development is based solely on cell size. My concern is not what to call these cells (whether "MMC-like" or "enlarged hypodermal"), but that it is not possible to be confident the ectopic large cells have anything to do with the hypodermal/MMC lineage at all. There are no other signs of hypodermal or MMC differentiation, and as the other reviewer points out the large cells do not even appear to have the large nucleolis that is characteristic of MMCs and their precursors. What data rules out a model where they are actually enlarged somatic cells? As it stands, I think the conclusion "double mutant drm1drm2 also presents supernumerary MMC-like cells, confirming the role of the RdDM pathway in the MMC formation" is overstated and should be toned down to say "double mutant drm1drm2 also presents ectopic enlarged cells, consistent with supernumerary MMC-like cells." This is a big weakness because it is really the only direct link in the paper between the drm1drm2 mutant and MMC formation.

Overall, my recommendation would be to tone down claims that the ectopic large cells are early MMC precursors, as this cannot be convincingly shown. It is a reasonable conclusion and worth discussing, but other models would also be consistent with the data. I would leave it at the editor's discretion whether the manuscript fits with Development given this core finding is not conclusive. The authors have been very thorough and careful in their work and the remaining conclusions in the paper are now convincing. I would support publication of this manuscript despite this key shortcoming.

Comments for the author

Other minor comments:
-For germline terminology, I would argue that terms like "hypodermal cell" would be more accurate than "germline precursor" and would not lead to confusion... but the end goal is clarity and the authors have clearly thought about this. The final decision on this terminology should be made by the authors.

-In Figure 1, the authors analyze their data very carefully by having 3 independent scientist score the images without knowledge of the genotype (as mentioned in the reviewer response); however, I still cannot find where in the manuscript where this approach is mentioned. Given the authors went through the trouble to analyze these data so carefully, it would strengthen the manuscript to make sure this is clear in the text. I must just be missing where this is described, but perhaps adding a few words in the figure legend or results would make it easier to find?

© 2020. Published by The Company of Biologists under the terms of the Creative Commons Attribution License (https://creativecommons.org/licenses/by/4.0/).

Reviewer 2

Advance summary and potential significance to field

This revised version of the manuscript by Mendes et al mostly answers the criticisms that I had with the previous version.

Comments for the author

Overall, I think they did a good job by analyzing earlier stages of ovule development, adding images of SPL_GFP during meiotic stages, and addressing reviewer 1 comments pertaining to statistical tests. I would thus recommend publication of the manuscript in its current form.

Second revision

Author response to reviewers' comments

Answer to the reviewers

Reviewer 1 Advance Summary and Potential Significance to Field:
The revised version of this manuscript is much improved. In particular, the quantification and statistical analysis of the data is more complete and stronger; areas that were insufficiently analyzed or unclear have largely been corrected. The new imaging of the SPL- GFP fusion is a beautiful addition and made it much easier to see the expression pattern of this construct.

We appreciate the reviewer's comment very much and are appreciative of the useful suggestions that have led us to improve the manuscript.

Currently, the key weakness in the previous version still remains. Specifically, the core phenotype linking the drm1drm2 double mutant to hypodermal/MMC development is based solely on cell size. My concern is not what to call these cells (whether "MMC- like" or "enlarged hypodermal"), but that it is not possible to be confident the ectopic large cells have anything to do with the hypodermal/MMC lineage at all. There are no other signs of hypodermal or MMC differentiation, and as the other reviewer points out the large cells do not even appear to have the large nucleolis that is characteristic of MMCs and their precursors. What data rules out a model where they are actually enlarged somatic cells? As it stands, I think the conclusion "double mutant drm1drm2 also presents supernumerary MMC-like cells, confirming the role of the RdDM pathway in the MMC formation" is overstated and should be toned down to say "double mutant drm1drm2 also presents ectopic enlarged cells, consistent with supernumerary MMC-like cells." This is a big weakness because it is really the only direct link in the paper between the drm1drm2 mutant and MMC formation.

We thank the reviewer for this comment. As suggested we have changed the sentence in the abstract.
We would like to emphasise that the extra enlarged cells found in *drm1drm2* double mutant in and in other reported *RdDM* mutants are link to the SPL/NZZ ectopic expression in the L1 layer of the ovule primordia.
With regard to the identification of enlarged nucleoli that was mentioned by reviewer 2, we have now provided new imaging in figure 1 and a new supplementary image in figure S1 where the typical MMC large nucleoli are identifiable in the extra enlarged cells. Reviewer 2 seems to agree with our observations.

Overall, my recommendation would be to tone down claims that the ectopic large cells are early MMC precursors, as this cannot be convincingly shown. It is a reasonable conclusion and worth discussing, but other models would also be consistent with the data. I would leave it at the editor's discretion whether the manuscript fits with Development given this core finding is not conclusive. The authors have been very thorough and careful in their work and the remaining conclusions in the

© 2020. Published by The Company of Biologists under the terms of the Creative Commons Attribution License (https://creativecommons.org/licenses/by/4.0/).

paper are now convincing. I would support publication of this manuscript despite this key shortcoming.

We appreciate the support for publication demonstrated by this reviewer.
As suggested, we have tried to improve the logic and 'readability' of the conclusions, and have changed the subheadings where appropriate.

Results:
We have changed
-Ectopic expression of *SPL/NZZ* induces supernumerary female germline precursors-
To
Ectopic expression of *SPL/NZZ* induces multiple MMC-like cells-

Discussion:

We have changed
-Methylation via *RdDM* is required for a single germline precursor –
To –
Absence of Methylation via *RdDM* leads to a multiple MMC-like cell phenotype

Reviewer 1 Comments for the Author:
Other minor comments:
-For germline terminology, I would argue that terms like "hypodermal cell" would be more accurate than "germline precursor" and would not lead to confusion... but the end goal is clarity and the authors have clearly thought about this. The final decision on this terminology should be made by the authors.

We thank the reviewer for this suggestion. However, we prefer to use "germline precursor" for, as we have explained earlier this term is more readily understood by a general 'developmental biology' audience, and, importantly, we only use it when discussing the model.

-In Figure 1, the authors analyze their data very carefully by having 3 independent scientist score the images without knowledge of the genotype (as mentioned in the reviewer response); however, I still cannot find where in the manuscript where this approach is mentioned. Given the authors went through the trouble to analyze these data so carefully, it would strengthen the manuscript to make sure this is clear in the text. I must just be missing where this is described, but perhaps adding a few words in the figure legend or results would make it easier to find?
We thank the reviewer for the suggestion, we have added now a sentence in the material and methods section.
We hope that the answers and changes we have made satisfy this reviewer, and- as always – we are always happy to clarify any other matters.

Reviewer 2 Advance Summary and Potential Significance to Field:
This revised version of the manuscript by Mendes et al mostly answers the criticisms that I had with the previous version.

Reviewer 2 Comments for the Author:
Overall, I think they did a good job by analyzing earlier stages of ovule development, adding images of SPL_GFP during meiotic stages, and addressing reviewer 1 comments pertaining to statistical tests. I would thus recommend publication of the manuscript in its current form.
We appreciate very much this reviewer's comments and thank him/her for the useful suggestions that have led us to improve the manuscript. We also appreciate this reviewer's support for the work in general.

© 2020. Published by The Company of Biologists under the terms of the Creative Commons Attribution License (https://creativecommons.org/licenses/by/4.0/).

Third decision letter

MS ID#: DEVELOP/2020/194274

MS TITLE: The RNA dependent DNA methylation pathway is required to restrict SPOROCYTELESS/NOZZLE expression to specify a single female germ cell precursor in Arabidopsis

AUTHORS: Marta A. Mendes, Rosanna Petrella, Edoardo Vignati, Stefano Gatti, Mara Cucinotta, Sara C. Pinto, Dayton C. Bird, Veronica Gregis, Hugh Dickinson, Matthew R. Tucker, and Lucia Colombo
ARTICLE TYPE: Research Article

I am happy to tell you that your manuscript has been accepted for publication in Development, pending our standard ethics checks.

Reviewer 1

Advance summary and potential significance to field

The revised version mostly addresses the comments I made, and conclusions that were over-stated have been edited to better reflect the data

Comments for the author

I would recommend publication in the current form and congratulate the authors on their work

Reviewer 2

Advance summary and potential significance to field

Overall, the authors have satisfactorily answered most of the criticisms raised by both reviewers.

Comments for the author

The images are now of much better quality, and I would suggest that it might be published in its current form.

© 2020. Published by The Company of Biologists under the terms of the Creative Commons Attribution License (https://creativecommons.org/licenses/by/4.0/).
